# A Novel *mcr-1* Variant Carried by an IncI2-Type Plasmid Identified From a Multidrug Resistant Enterotoxigenic *Escherichia coli*

**DOI:** 10.3389/fmicb.2018.00815

**Published:** 2018-04-25

**Authors:** Hongbo Liu, Binghua Zhu, Beibei Liang, Xuebin Xu, Shaofu Qiu, Leili Jia, Peng Li, Lang Yang, Yongrui Li, Ying Xiang, Jing Xie, Ligui Wang, Chaojie Yang, Yansong Sun, Hongbin Song

**Affiliations:** ^1^College of Military Medicine, Academy of Military Medical Sciences, Beijing, China; ^2^Institute of Disease Control and Prevention, People’s Liberation Army, Beijing, China; ^3^Shanghai Municipal Centre for Disease Control and Prevention, Shanghai, China

**Keywords:** mobilized colistin resistance (*mcr-1*) gene, novel *mcr-1* variant, clinical diarrhea, enterotoxigenic *Escherichia coli* (ETEC), multidrug resistance (MDR)

## Abstract

In this study, we discovered a novel mobilized colistin resistance (*mcr-1*) gene variant, named *mcr-1.9*, which was identified in a colistin-resistant enterotoxigenic *Escherichia coli* (ETEC) strain from a clinical diarrhea case. The *mcr-1.9* gene differs from *mcr-1* at position 1036 due to a single nucleotide polymorphism (G→A), which results in an aspartic acid residue being replaced by an asparagine residue (Asp346→Asn) in the MCR-1 protein sequence. Antimicrobial susceptibility testing showed that the *mcr-1.9*-harboring ETEC strain is resistant to colistin at a minimum inhibitory concentration of 4 μg/ml. Plasmid profiling and conjugation experiments also suggest that the *mcr-1.9* variant can be successfully transferred into the *E. coli* strain J53, indicating that the gene is located on a transferable plasmid. Bioinformatics analysis of data obtained from genome sequencing indicates that the *mcr-1.9* gene is located on a 64,005 bp plasmid which has been named pEC26. This plasmid was found to have high similarity to the *mcr-1*-bearing IncI2-type plasmids pWF-5-19C (99% identity and 99% coverage) and pmcr1-IncI2 (99% identity and 98% coverage). The *mcr-1.9-*harboring ETEC also shows multidrug resistance to nine classes of antibiotics, and contains several virulence and antimicrobial-resistance genes suggested by the genome sequence analysis. Our report is the first to identify a new *mcr-1* variant in an ETEC isolated from a human fecal sample, raising concerns about the existence of more such variants in human intestinal flora. Therefore, we believe that an undertaking to identify new *mcr-1* variants in the bacterial communities of human intestines is of utmost importance, and that measures need to be taken to control the spread of *mcr-1* and its variants in human intestinal microflora.

## Introduction

Diarrheal diseases are the second leading cause of death in children under 5 years of age (Qadri et al., 2005). Each year, diarrhea kills ∼525,000 children worldwide (World Health Organization [WHO], 2017). Enterotoxigenic *Escherichia coli* (ETEC) is an extremely important causative agent of diarrhea in the developing world, due to the prevalence of unhygienic conditions that include the unavailability of clean water and poor sanitation. It is the most frequently encountered bacterial agent causing diarrhea in both children and adults, as well as the most common cause of traveler’s diarrhea in these areas (von Sonnenburg et al., 2000; Bourgeois et al., 2016). ETECs are defined by their ability to produce one or both of two types of enterotoxins [heat-stable (ST) and heat-labile (LT) (So et al., 1978; Rao, 2008)], besides which, they may also produce several colonization factors (CFs) (So et al., 1978) and carry chromosomal determinants associated with ETEC virulence (Del Canto et al., 2011).

Antimicrobial resistance (AMR) has now been recognized as a significant global threat to human and animal health (World Health Organization [WHO], 2018). Due to the widespread and indiscriminate use of antibiotics in countries where diarrhea is endemic, the rate of multidrug resistance (MDR, resistance to three or more classes of antimicrobials) in *Enterobacteriaceae* such as ETEC, is increasing alarmingly (Lynch et al., 2013). So far, due to this increase in MDR, the usage of successively stronger antimicrobial agents belonging to the tetracycline, sulfonamide, macrolide, and fluoroquinolone families to treat ETEC infections has increased (Diemert, 2006; Rodas et al., 2011). Fluoroquinolones are one of the most recently developed, powerful, and broad-spectrum antibiotics used for treating a wide variety of bacterial infections (Oethinger et al., 1996). Unfortunately, since their introduction in the late 1980s, bacterial resistance to fluoroquinolones has increased markedly (Aguiar et al., 1992). Colistin, which is a polymyxin antibiotic, is currently used as a last-resort antibiotic for treating infections caused by Gram-negative bacteria exhibiting MDR. However, a study conducted by Liu et al. (2015) on *E. coli* strains from China, has described a new plasmid-mediated colistin-resistance mechanism conferred by the phosphoethanolamine transferase-encoding gene *mcr-1* (Liu et al., 2015). Since then, numerous studies on *mcr-1* have been undertaken worldwide to investigate the presence of this gene in *E. coli* strains isolated from various animals, environments, food samples, and even human subjects (Doumith et al., 2016; Khalifa et al., 2016; Perrin-Guyomard et al., 2016; Suzuki et al., 2016; Zhao et al., 2017a).

Since the discovery of *mcr-1*, several variants of the gene, such as *mcr-1.2, mcr-2, mcr-1.3, mcr-1.4, mcr-1.5, mcr-1.6, mcr-1.7*, and *mcr-1.8* have been discovered in Italy, Belgium, China, and Brunei (Di Pilato et al., 2016; Xavier et al., 2016; Lu et al., 2017; Tijet et al., 2017; Yang et al., 2017; Zhao et al., 2017b). The variants, *mcr-2, mcr-1.3, mcr-1.4, mcr-1.5, mcr-1.7*, and *mcr-1.8* are known to have been identified from *E. coli* strains isolated from animal, sewage, or human urinary tract samples (the source for *mcr-1.8* has not been indicated) (**Table 1**). In this study, we report a new variant of the *mcr-1* gene, which we term *mcr-1.9*, identified from an *E. coli* strain (an ETEC) isolated from a diarrheal fecal sample. Apart from analyzing the molecular and AMR characteristics of this ETEC strain, we have also identified the plasmid type in which *mcr-1.9* is located, and compared it with other similar *mcr-1*-harboring plasmids described elsewhere. We believe that this is the first report that identifies a new *mcr-1* variant from an ETEC strain isolated from a human fecal sample.

**Table 1:** List of currently known *mcr-1* gene variants and *mcr-2*.

Variants	Accession numbers	Nucleotide mutations	Amino acid variations	Host strain	Source	Country	Reference
*mcr-1*	KP347127	–	–	*E. coli*	Pig	China	Liu et al., 2015
*mcr-2*	LT598652	–	–	*E. coli*	Calves	Belgium	Xavier et al., 2016
*mcr-1.2*	KX236309	A8T	Q3L	*K. pneumoniae*	Human rectal swab	Italy	Di Pilato et al., 2016
*mcr-1.3*	KU934208	A111G, A112G	I38V	*E. coli*	Chicken	China	Yang et al., 2017
*mcr-1.4*	KY041856	G1318T	D439N	*E. coli*	Sewage	China	Zhao et al., 2017b
*mcr-1.5*	KY283125	C1354T	H452Y	*E. coli*	Human urine	Argentina	Tijet et al., 2017
*mcr-1.6*	KY352406	A1263G, A1607G	T215A, R536H	*S. Typhimurium*	Human rectal swab	China	Lu et al., 2017
*mcr-1.7*	KY488488	G643A	A215T	*E. coli*	Sewage	China	Zhao et al., 2017b
*mcr-1.8*	KY683842	A8G	Q3R	*E. coli*	–	Brunei	–
*mcr-1.9*	MG946761	G1036A	D346N	*E. coli*	Human fecal	China	This study

## Materials and Methods

### Bacterial Isolation, Serotyping, and *mcr-1* Gene Screening

A fecal sample was obtained from a 15-month-old male child diagnosed with watery diarrhea and fever, cured with ceftriaxone in 2014 at the Shanghai Children’s Hospital, China. The fecal sample was streaked onto MacConkey agar and incubated overnight at 37°C. Colonies having a pink coloration and a round, neat appearance were selected as suspected *E. coli* colonies. These were again streaked directly onto MacConkey agar, and incubated overnight at 37°C, following which, the colonies so obtained were further sub-cultured on Luria–Bertani (LB) agar plates at 37°C. Genomic DNA was prepared using the boiling method from the isolated bacteria, and a Diarrhoeagenic *E. coli* (DEC) detection PCR kit (SSI Diagnostica, Denmark) was used to identify the type of *E. coli* strain obtained from the fecal sample. The PCR reactions were performed in a 20-μl volume containing 10 μl of PCR ReadyMix, 6 μl of Primer mix, and 4 μl of DNA template. The cycling conditions were 95°C for 2 min, followed by 35 cycles of denaturation at 94°C for 50 s, annealing at 62°C for 40 s, extension at 72°C for 50 s, and a final extension at 72°C for 3 min in an Applied Biosystems PCR cycler. The PCR products are then loaded into 2% agarose gel and electrophoresis. The type of DEC was then identified by the comparison on the fragment size of PCR products and marker in the kit. We screened for the presence of the *mcr-1* gene in this isolate (termed SH66) without prior knowledge of its colistin-resistance. The screening was carried out with PCR and Sanger sequencing using previously described primers (Liu et al., 2016).

Informed and written consents were obtained from the patient’s parents for sample collection and usage, as well as for the publication of obtained results in this study. All experiments were approved and authorized by the Ethics Committees of the Institute of Disease Prevention and Control, People’s Liberation Army, China.

### Antimicrobial Susceptibility Testing

Minimum inhibitory concentrations (MICs) of colistin for all isolates obtained from the fecal sample were determined using the 96-well plate dilution method, with a maximum colistin concentration of 32 μg/mL, and a minimum concentration of 0.5 μg/mL; the results were interpreted according to the conventions set by the European Committee on Antimicrobial Susceptibility Testing (EUCAST)^1^. For the *mcr-1*-positive isolates, we also tested for antimicrobial susceptibility to other antimicrobials via the broth microdilution method using a 96-well microtiter plate (Sensititre, Trek Diagnostic Systems, Thermo Fisher Scientific Inc., West Sussex, United Kingdom). MICs were determined according to the clinical and laboratory standards institute (CLSI, 2014) recommendations (Bennett et al., 2014). The *E. coli* strain ATCC 25922 was used for quality control.

### Plasmid Profiling and Southern Blotting

The DNA of ETEC strain for plasmid profiling was obtained and embedded into agarose according to the pulsed-field gel electrophoresis (PFGE) plug preparation method (Ribot et al., 2006). Then the plug was cut into slices and treated with S1 endonuclease, following which, the DNA fragments were subjected to PFGE according to the PulseNet standard protocols (Ribot et al., 2006). Following this, the membrane transfer, molecular hybridization, and probe detection steps were performed according to a previously reported method (Zou et al., 2015). The *mcr-1* probe sequences used in the southern blotting analysis were identical to the *mcr-1* primer sequences mentioned in Section “Bacterial Isolation, Serotyping, and *mcr-1* Gene Screening.”

### Conjugation Experiments

Conjugation experiments to test the transferability of the *mcr-1*-positive plasmid were carried out using the *E. coli* strain J53 (LacZ^-^, AzrR, RifR) as a recipient, and the *mcr-1*-positive *E. coli* strain (identified in the fecal sample and named SH66) as a donor. Overnight cultures of donor and recipient bacterial strains (3 mL of each) were mixed together, harvested, and resuspended in 80 μl of LB with a donor:recipient ratio of 1:3. The resultant mixture was spotted into 5 mL of liquid LB broth and incubated 37°C for 12 to 18 h for mating to occur. Following this, a small amount of the mating mixture was spotted on to a Muller-Hinton agar plate (BD Biosciences, San Jose, CA, United States) containing 100 mg/L sodium azide and 2 mg/L polymyxin B; this was used as a selective medium to identify *E*. *coli* J53 transconjugants. The identities of putative transconjugants were confirmed using antimicrobial susceptibility testing and PCR detection of the *mcr-1.9* gene as described before. The conjugation transfer rate was calculated through the formula “R = number of transconjugants/number of recipients.”

### Whole Genome Sequencing and Bioinformatic Analysis

The genomic DNA of the *E. coli* isolate SH66 was extracted using the QIAamp DNA Mini Kit (Qiagen, Germany), and sequenced using the Illumina MiSeq platform (Illumina Inc., San Diego, CA, United States) according to the manufacturer’s instructions. A DNA library was constructed with inserts of length 600 bp using the MiSeq Reagent Kit v2. The raw sequence data were assembled *de novo* via the CLC Genomics Workbench 9.0.1 (CLC Bio, Qiagen Bioinformatics, Germany). The *mcr-1* plasmid sequence was assembled through the above software and the gaps were filled through combinatorial PCR and Sanger sequencing. The point mutation in *mcr-1.9* was verified by the PCR amplification and Sanger sequencing with the primers MCR1-3-F (5′-GGCGTATTCTGTGCCGTGTA-3′) and MCR1-3-R (5′-CGTGATCGCGTCATGGGT-3′) (**Supplementary Data Sheet S2**). Resistance genes, virulence genes, and other genetic structures were identified through the center for genomic epidemiology (CGE) website^2^. Sequence alignments were performed using the software Mauve (Darling et al., 2004), and further rounds of PCR amplification and Sanger sequencing were used to fill in the gaps. Complete plasmid sequences were annotated using the online annotation server RAST (Aziz et al., 2008). The plasmid replicons, IS sequences, and AMR genes were identified using the online software PlasmidFinder^3^, ISfinder^4^, and ResFinder^5^, respectively. Sequence comparisons and map generation were performed using BLAST^6^ and Easyfig (Sullivan et al., 2011), respectively.

## Results

### *mcr-1* Gene Screening and Antimicrobial Susceptibility Testing

One *E. coli* strain (termed SH66) was isolated from the fecal sample used in this study, and identified as an ETEC using the DEC PCR detection kit. This strain was subsequently transferred to and preserved in our laboratory, where it was recently screened for the presence of the *mcr-1* gene with no prior knowledge of its colistin-resistance characteristic. The screening indicated that this strain harbored an *mcr-1* gene. According to medical records, the patient who harbored this strain was not treated with polymyxin antibiotics.

The results of the antimicrobial susceptibility testing indicate that the ETEC strain identified in this study is resistant to colistin (MIC, 4 μg/ml), ampicillin (>32 μg/ml), chloramphenicol (>32 μg/ml), trimethoprim/sulfamethoxazole (>4 μg/ml), tetracycline (>32 μg/ml), ciprofloxacin (>4 μg/ml), sulfonazo isoxazole (>256 μg/ml), nalidixic acid (>32 μg/ml), streptomycin (>64 μg/ml), and azithromycin (>16 μg/ml), though it was found to be susceptible to amoxicillin/clavulanic acid (4 μg/ml), ceftriaxone (≤0.25 μg/ml), ceftiofur (0.25 μg/ml), cefoxitin (8 μg/ml), and gentamicin (1 μg/ml) (**Table 2**). According to the classification standards of the WHO (Collignon et al., 2009), SH66 is an MDR strain showing resistance to nine classes of antimicrobials, including aminoglycosides, amphenicols, folate pathway inhibitors, macrolides, penicillins, polymyxins, quinolones, sulfonamides, and tetracyclines.

**Table 2:** Comparisons of antimicrobial resistance levels between the donor *E. coli* strain SH66, recipient strain J53 and the J53 transconjugants.

Antimicrobial class	Antimicrobial	Donor strain SH66		Transconjugants J53		Recipient strain J53	
		Susceptibility	MICs	Susceptibility	MICs	Susceptibility	MICs
Polymyxins	PolymyxinB/Colistin	R	4	R	4	S	0.125
Aminoglycosides	Gentamicin	S	1	S	≤0.25	S	≤0.25
	Streptomycin	R	>64	R	>64	S	≤2
Amphenicols	Chloramphenicol	R	>32	R	>32	S	≤8
Cephalosporins	Ceftriaxone	S	≤0.25	S	≤0.25	S	≤0.25
	Ceftiofur	S	0.25	S	≤0.5	S	≤0.5
Cephamycins	Cefoxitin	S	8	S	≤8	S	≤8
Folate pathway inhibitors	TRI/SUL	R	>4	R	>4	S	≤2
Macrolides	Azithromycin	R	>16	R	>16	S	≤4
Penicillins	AMO/CLA	S	4	S	≤4	S	≤4
	Ampicillin	R	>32	R	>32	S	≤8
Quinolones	Ciprofloxacin	R	>4	S	≤0.015	S	≤0.015
	Nalidixic acid	R	>32	S	≤4	S	≤4
Sulfonamides	Sulfisoxazole	R	>256	R	>256	S	≤16
Tetracyclines	Tetracycline	R	>32	R	>32	S	≤4

*The classifications of antimicrobial agents are based on the classification standards of WHO (World Health Organization) (Collignon et al., 2009). The unit of MICs isμg/ml. S, susceptible; R, resistant; TRI/SUL, trimethoprim/sulfamethoxazole; AMO/CLA, amoxicillin/clavulanate.*

### Conjugation Experiments and Southern Blotting

In order to determine the transferability of the *mcr-1* gene carried by SH66, conjugation experiments were carried out using the *E. coli* strain J53 as a recipient, and SH66 as the donor strain. All transconjugants obtained in this experiment tested positive for the *mcr-1* gene via PCR amplification and sequencing analysis. The conjugation transfer rate is 1.6 × 10^-5^. Colistin susceptibility testing showed that the MIC values for the transconjugants (the J53 strains carrying the *mcr-1* gene) increased to 4 μg/ml (**Table 2**). This is much higher than the colistin resistance levels of the original J53 strains (which have MIC values of 0.125 μg/ml), and indicates that the transconjugants acquire the same colistin resistance gene as the donor strain.

The S1 endonuclease-PFGE and southern blotting analysis revealed that the *mcr-1* gene in SH66 was located on a plasmid approximately 60 kb in length (**Supplementary Figure S1**).

### Virulence and Resistance Factors Carried by the *mcr-1*-Harboring ETEC Strain SH66

The DNA of the *E. coli* strain SH66 identified in this study were subjected to 600-bp paired-end whole genome sequencing with 73.4-fold coverage using the MiSeq sequencer. A total of 1,364,690 reads and 405,565,025 clean bases were generated, which were assembled into 107 contigs with an N50 length of 216,913 bp. Bioinformatics analyses showed that this strain carries the virulence genes *eltA, eltB, astA, eilA, lpfA, ltcA*, and *stb*. Additionally, SH66 also carries several resistance genes, namely, *bla*_TEM-1_, *mphA, ermB, aph(3’)-Ia, aadA5, strA, strB, sul1, sul2, tetA, tetM, floR, catA1, dfrA17, oqxA*, and *oqxB*, along with the *mcr-1* gene, all of which could mediate resistance to β-lactams, macrolides, aminoglycosides, sulphonamides, tetracyclines, phenicols, trimethoprim, quinolones, and colistin (**Supplementary Table S1**). The presence of these genes also explains the increase in resistance to several antibiotics observed in the *E. coli* J53 transconjugants after the conjugation experiment (**Table 2**). Analyses of the *gyrA* and *parC* point mutations carried by SH66 also showed that *gyrA* contained the Ser83→Leu and Asp87→Asn substitutions, along with the *parC* mutations Ser57→Thr and Ser80→Ile.

### Identification and Analysis of the Plasmid Carrying the Novel Variant, *mcr-1.9*

An *mcr-1*-carrying plasmid of length 64,005 bp, which we have named pEC26, was identified in SH66 through sequence analysis. Interestingly, the *mcr-1* gene identified on this plasmid was found to be a variant of the original *mcr-1* due to an SNP (**Supplementary Figure S2**). The SNP was located at position 1036 (where a G→A transition had occurred), which results in the replacement of an aspartic acid residue with an asparagine residue (Asp346→Asn). Since the position of this SNP is different from those of all previously discovered *mcr-1* variants, we named this variant *mcr-1.9*. The sequence analysis shows that there is no resistance gene in addition to *mcr-1.9* located in plasmid pEC26.

**FIGURE 1:**
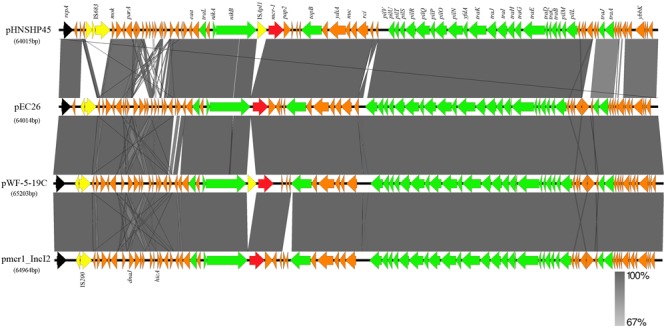
Linear comparisons of complete sequences of the IncI2-type plasmids pHNSHP45 (accession no. KP347127), pmcr1_IncI2 (KU761326), pWF-5-19C (KX505142), and pEC26 (MG946761). Colored arrows represent open reading frames, with black, yellow, green, red, and orange arrows representing replication genes, mobile elements, plasmid transfer genes, the *mcr-1* gene, and plasmid backbone genes, respectively. Gray shading denotes regions of shared homology amongst the different plasmids.

**FIGURE 2:**
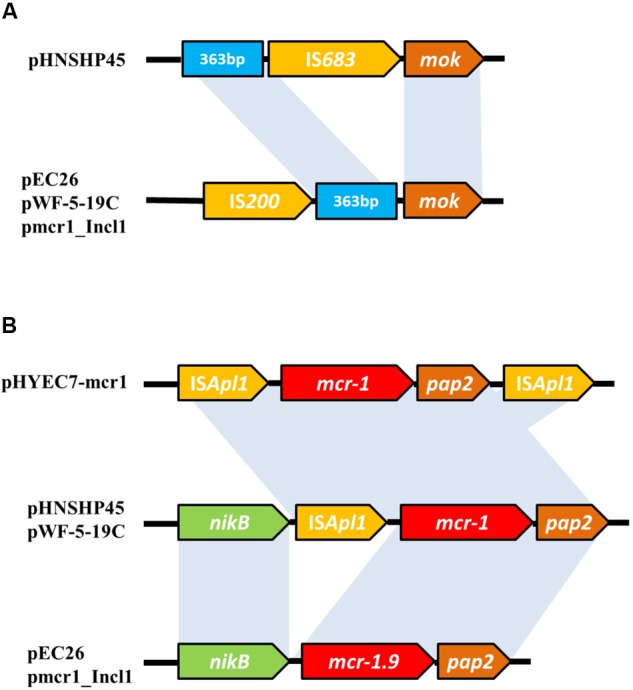
A comparison of the genetic environments of IS683 and IS200 **(A)**, along with *mcr-1* and *mcr-1.9* **(B)** in the plasmids pHYEC7-mcr1 (Li et al., 2017), pHNSHP45, pWF-5-19C, pmcr1_Incl1 and pEC26. Gray shading indicates >99% nucleotide identity.

The plasmid pEC26 belongs to the IncI2 group, the same plasmid incompatibility group as pHNSHP45, which was the first plasmid reported to harbor *mcr-1* (Liu et al., 2015); pEC26 also harbors 86 predicted open reading frames (ORFs), and has a GC content of 42.55% with a plasmid backbone very similar to that of other IncI2-type plasmids, such as pmcr1-IncI2 (99% identity and 98% coverage, GenBank accession no. KU761326) (Li et al., 2016) and pWF-5-19C (99% identity and 99% coverage, KX505142) (Liu et al., 2017). A comparison of these plasmids and pHNSHP45 (KP347127) (Liu et al., 2015) shows that pEC26, pmcr1-IncI2, and pWF-5-19C contain an IS200 element before the *mok* gene instead of the IS683 found in pHNSHP45. However, the IS200 element in these plasmids is inserted before a 363 bp fragment that precedes the IS683 element in pHNSHP45 (**Figures 1, 2A**). This indicates that although these two IS elements are inserted into the same plasmid backbone, their positions are not the same. Like all the other plasmids mentioned here, pEC26 also contains the 2,607 bp DNA segment that includes the *mcr* (*mcr-1* or *mcr-1.9*) and *pap2* genes, which are integrated downstream of the *nikB* gene. Besides this, the *mcr-1*-associated IS*Apl1* element was not found in the pEC26 plasmid (**Figures 1, 2B**). The sequence of pEC26 (**Supplementary Data Sheet S1**) has been deposited with GenBank under the accession number MG946761.

## Discussion

This study reports the identification of a novel *mcr-1* gene variant, *mcr-1.9*, which was harbored in an ETEC strain isolated from the diarrheal fecal sample of a 15-month-old male child. We find that the variant *mcr-1.9*, differs from the *mcr-1* gene at position 1036 (where a G→A transition had occurred), which results in the replacement of an aspartic acid residue with an asparagine residue (Asp346→Asn). The MCR-1 protein consists of a transmembrane domain and a soluble catalytic domain. The transmembrane domain is located on the N-terminal, and is composed of residues 1–214, whereas the soluble catalytic domain is located on the C-terminal, and is composed of residues 215–541 (Stojanoski et al., 2016). Therefore, the Asp346→Asn change detected in *mcr-1.9* is located in the catalytic domain. Since four of the known *mcr-1* gene variants, *mcr-1.4–1.7*, also contain point mutations in the catalytic domain (**Table 1**), it appears that this domain has a higher mutation rate than the transmembrane domain. However, although the currently identified mutations in the *mcr-1* gene have so far not showed obvious increases in colistin resistance, the presence of two or more point mutations in AMR genes can occasionally lead to changes in the strength of resistance imparted by the gene, such as the resistance increase to fluoroquinolones by the additional mutations in the *gyrA* and/or *parC* genes (Vila et al., 1994; Ruiz et al., 2002).

Apart from mutations causing heightened resistance to known antimicrobials, another concern regarding resistance genes is their spread through bacterial populations via horizontal transfer. The occurrence of the *mcr-1* variant *mcr-1.9* in an ETEC strain isolated from a human fecal sample is a cause of much concern; as the human intestine contains a complex collection of microflora where the incidence of horizontal gene transfer can occur at a high rate, the spread of this gene can cause widespread resistance to colistin. Added to this, several studies have shown that the *mcr-1* gene can be ubiquitously disseminated in the *Enterobacteriaceae* (Du et al., 2016; Malhotra-Kumar et al., 2016; Yang et al., 2016). Furthermore, the *mcr-1* gene is usually associated with the highly active transposon element *ISApl1* that can occur either on one, or both sides of the gene (Li et al., 2017; Snesrud et al., 2017) (**Figure 2B**). The presence of *ISApl1* could further enhance the probability of this gene being transferred horizontally; *in vitro* mobilization assays have already proved that the *mcr-1* gene can be mobilized by *ISApl1*-mediated transposition (Poirel et al., 2017). Moreover, the transfer of *mcr-1* via *ISApl1*-mediated transposition has been shown to generate mutations, leading to the formation of new *mcr-1* gene variants (Poirel et al., 2017). We therefore hypothesize that *mcr-1.9* and perhaps other variants of *mcr-1* may have been generated during horizontal transfer events involving transposable elements.

The first reported *mcr-1* gene was identified in an IncI2 plasmid (Liu et al., 2015). As of now, the *mcr-1* gene and its variants have been reported in various plasmid backbones, including the IncI2, IncHI2, IncF, IncP, IncFIP, and IncX4 (Liu et al., 2015; Doumith et al., 2016; Falgenhauer et al., 2016; Nordmann et al., 2016), all of which are known to be vehicles disseminating antibiotic resistance genes among the *Enterobacteriaceae.* The *mcr-1.9* gene identified by us was found on an IncI2-type plasmid, which has a broad host range (Lv et al., 2013). Furthermore, three [*mcr-1.3* (KU934208), *mcr-1.5* (KY471308), and *mcr-1.7* (KY829117)] of the six *mcr-1* variants [*mcr-1.8* (KY683842) has no description of plasmid type] previously discovered are located on IncI2-type plasmids. Besides this, the plasmid most similar to pEC26 is pWF-5-19C (KX505142), which is found in *Cronobacter sakazakii* isolated from chicken (Liu et al., 2017). Based on this information, we speculate that pEC26 and plasmids similar to it have a strong ability to facilitate horizontal transfer of the *mcr-1* gene, and that this gene may have a higher mutation rate when carried by IncI2-type plasmids than when they are present in other plasmid types. However, since only a few such instances have been identified so far, this hypothesis needs much further study for verification and confirmation.

Besides harboring the novel *mcr-1* gene variant, *mcr-1.9*, the ETEC that we have isolated and described in this study was found to carry various virulence genes, namely, heat-stable enterotoxin (*estA*), heat-labile enterotoxin (*eltB*), EAST1 (enteroaggregative *E. coli* heat-stable toxin 1) gene (*astA*), adherence related gene (*eilA*), long polar fimbriae (*lpfA*), heat-labile enterotoxin A subunit (*ltcA*), and heat-stale enterotoxin II (*stb*). The strain was also found to carry several antimicrobial-resistance genes and shows MDR to at least nine classes of antibiotics (**Table 2**). These results indicate that this strain, called SH66, probably has high AMR and pathogenicity. Since *mcr-1* variants have begun to appear in human intestinal bacteria, especially in diarrheal *E. coli* strains, it is very possible that the spread of this gene in human intestinal microflora may cause issues in the effective treatment of diarrheal conditions induced by bacterial infections.

Overall, our findings highlight the possibility of a widespread dissemination of *mcr-1* gene variants, along with various virulence factors and resistance genes in the bacterial flora of the human intestine. Therefore, we believe that it is of utmost importance to track and identify mutations in the *mcr-1* gene, and to control its transmission. We also believe that the judicious use of antibiotics by physicians is crucial to reduce the rate of emergence of MDR bacterial strains.

## Author Contributions

HS, YS, and CY designed the experiments. HL and BZ performed the experiments. SQ, YX, LJ, and BL analyzed the data. PL, YL, and LY prepared the tables and figures. CY, YX, JX, and LW prepared the manuscript. All authors read and approved the final manuscript.

## Conflict of Interest Statement

The authors declare that the research was conducted in the absence of any commercial or financial relationships that could be construed as a potential conflict of interest.

## Footnotes

1 http://www.eucast.org/clinicalbreakpoints/

2 http://www.genomicepidemiology.org/

3 https://cge.cbs.dtu.dk/services/PlasmidFinder/

4 https://www-is.biotoul.fr/search.php

5 https://cge.cbs.dtu.dk/services/ResFinder/

6 http://blast.ncbi.nlm.nih.gov

## Supplementary Material

FIGURE S1 Plasmid profiling and southern blotting of SH66. The red box marks the plasmid pEC26. The genome DNA of *Salmonella* ser. Braenderup H9812 were restriction digested with *Xba*I enzyme and the products were used as the markers. The sizes of products are showed on the left with the unit “kb.”

FIGURE S2 Nucleotide and protein sequence alignments of the *mcr-1* gene with its different variants. Only the alignment blocks carrying mutations found in *mcr-1.9* are shown. The mutations in the base and amino acid sequences are denoted by the use of a different color. In *mcr-1.9*, G was mutated to A in nucleotide position 1036, leading to the replacement of an Aspartic acid residue with an asparagine residue at amino acid position 346.

TABLE S1 List of resistance and virulence factors/genes found in the *mcr-1.9*-harboring ETEC identified in this study.

DATA SHEET S1 The complete sequence of *mcr-1.9* harboring plasmid pEC26 (GenBank accession number is MG946761).

DATA SHEET S2 The verification result files of the point mutation in *mcr-1.9* by the PCR amplification and Sanger sequencing.
