# Supplementary figures and images for: A Novel mcr-1 Variant Carried by an IncI2-Type Plasmid Identified From a Multidrug Resistant Enterotoxigenic Escherichia coli
Source: Front Microbiol. 2018 Apr 25;9:815. doi: 10.3389/fmicb.2018.00815 (PMC5996929; doi:10.3389/fmicb.2018.00815)

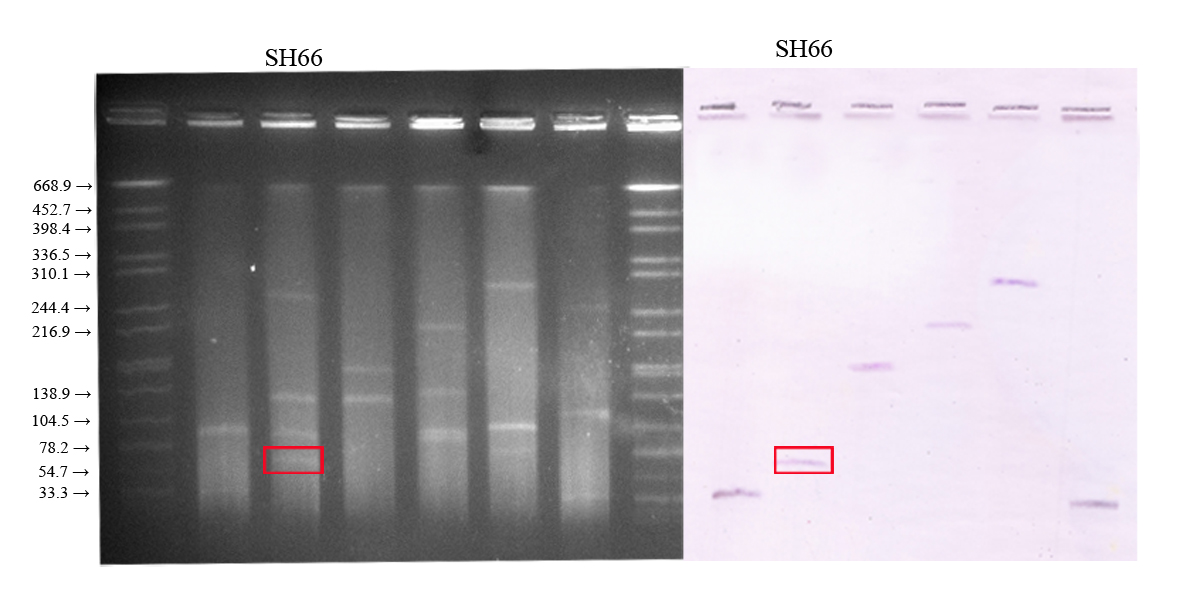

Supplement: FIGURE S1 — Plasmid profiling and southern blotting of SH66. The red box marks the plasmid pEC26. The genome DNA of Salmonella ser. Braenderup H9812 were restriction digested with XbaI enzyme and the products were used as the markers. The sizes of products are showed on the left with the unit “kb.” [file Image_1.JPEG]

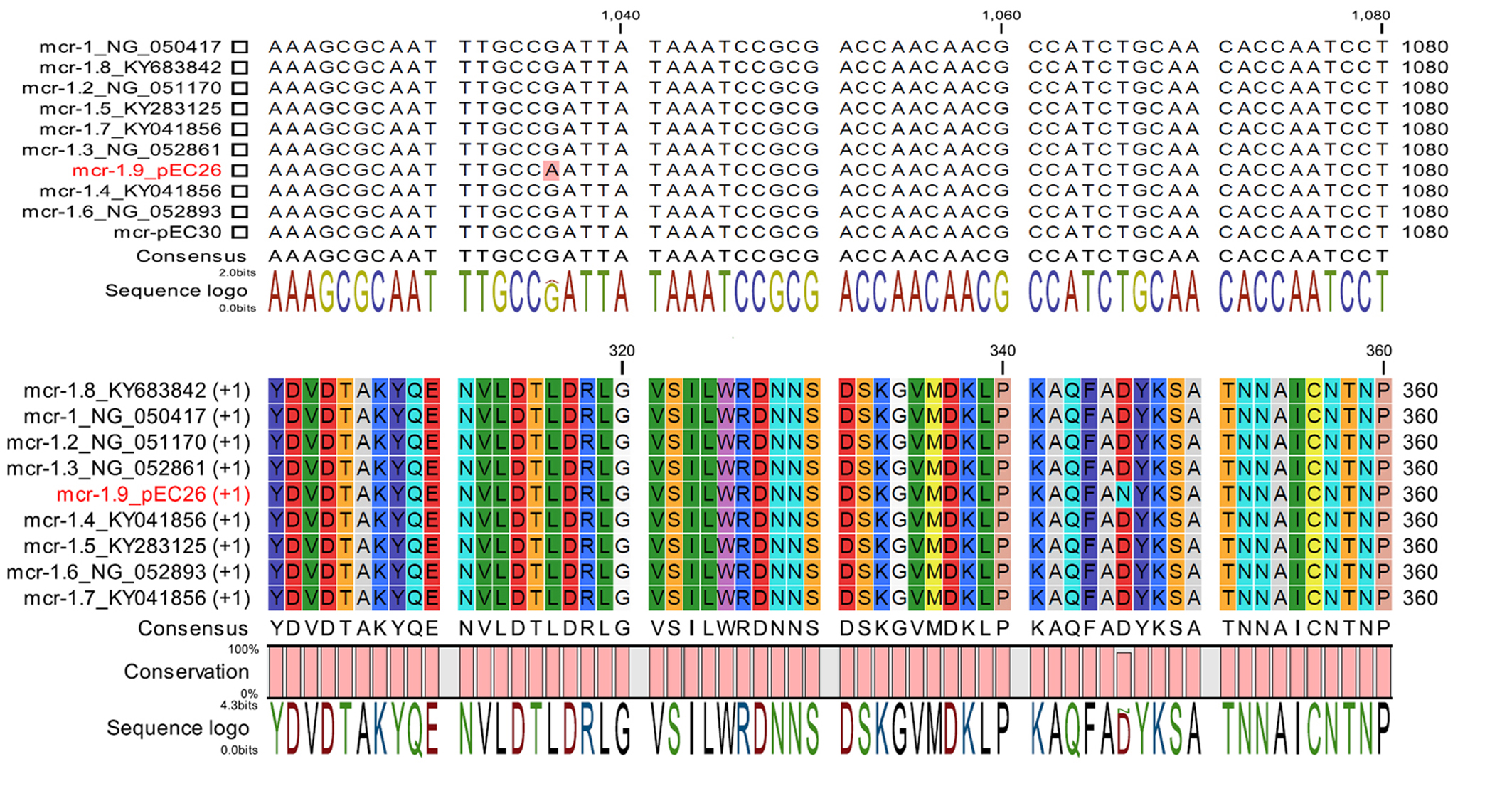

Supplement: FIGURE S2 — Nucleotide and protein sequence alignments of the mcr-1 gene with its different variants. Only the alignment blocks carrying mutations found in mcr-1.9 are shown. The mutations in the base and amino acid sequences are denoted by the use of a different color. In mcr-1.9, G was mutated to A in nucleotide position 1036, leading to the replacement of an Aspartic acid residue with an asparagine residue at amino acid position 346. [file Image_2.JPEG]
