# Supplementary material for: A Novel mcr-1 Variant Carried by an IncI2-Type Plasmid Identified From a Multidrug Resistant Enterotoxigenic Escherichia coli
Source: Front Microbiol. 2018 Apr 25;9:815. doi: 10.3389/fmicb.2018.00815 (PMC5996929; doi:10.3389/fmicb.2018.00815)
Supplement: DATA SHEET S1 — The complete sequence of mcr-1.9 harboring plasmid pEC26 (GenBank accession number is MG946761). [file Data_Sheet_1.DOCX]

>pEC26 Escherichia coli plasmid pEC26-mcr-1.9, complete sequence

CAGGCTTGAACATCGTTGATCGATTGCGCCCATGCTGCTAAATTTGCAGGCAAGTTTTCCGCGATTTGTAAATGCAGAAAACGAGGTGCTGAAATTTTGTTCGAGAACGTAAATTTCAGATAAAGAAAACCCCCGTAATCTCGTCTTTTCCAGGGAGCGCAGATTAGCGGGGGCTGACATCAAGGCAGAGCCTTGAGCTTATTTATTTCAGGTATGCTTGTTACCTGAGATGGAGTTTAATTCCATGAGAAGGAAATATCAACTCTTACAGTGCAAGCTAAGTGCAGCTTGCTGTGATTAGCAAACGTCACTGGTCTGAACTATCACCGGAAGAGCAGGTCCGTTTCTGGCAGGACTACGAAGCGGGTATTGAAAGCTCATTCCTGGTCCTCCAGGAAAACAAAGGCGGAACAACAAAACGTCGCCGGGGTGAACATTCCACTAAGCCAAAGTGTGAGAATCCAGCCTGGTTCCGTCCCGACAACTATAAGGCGCTGGGCGGACAGTTGGGCCACGCTTACAATCGTCTCGTTAAAAAAGACCCGGTCACTGGTCAGAACACACTCAGAATGCACATGTCGTTGCATCCATTTTATGTACTGAAACGTCAGAGTGTCGGTCGCAAATATAAATTCCGACCGGAAAAACAGCGGCTGCTTGACGCAATATGGGTTGTACTTGTGAGTTTTTGTGATCGTGGATTGCACACTGTCGGCATGTCTGTCTCCCGCCTGGCAGAAGAAATAAGCCCAAAAGACAGTAAGGGAGACGTGATTCCTGAAACCGCAGTCACAGTATCGCGCCTTTCCCGTCTGCTGGCAGAGCAGGTTTCCTTTGGCACGTTGGGAACGTCAGAAAAGACGATATGGGACCGTGAATCCCGCCAGCGACTGCCGAAATACGTCTGGATCACCGAAACTGGCTGGAAAATGCTGGGAGTTGATCTGGTGAAACTTCAGGAACAGCAACGTAAACGCCTTGCAGAAAGTGAAATCCGTCTGCAATTGATTAAAGAAGGCGTTATCCGCGAGGGCGAAGAAATCTCTGTTCACTCGGCTCGTAAACGCTGGTATGCGCAGCGTTCTCTCGATGCCATCAAGTCCCGACGTAAGAAAGCAGCGGAGCGCAAACGTGCTAATCGCCTGGCAAAACTGCCGTATGATGAACAGCGAAACGAAATTGCGCGGTTTATTCTGAAGCGTATGCCGCCGGACGAAGCGTACTGGTGCACTAAAGAAAGATTAGAGCAACTGGTAGCCAGAGATCTACGTCAGCTTGAACTGGCTCTGACAGCTTCGCCACCCCACTAGTTTAGCTGCCTGATACAATCGGCACTCTCTTCAGCCCCTTCGGGGCTTTTCGTGTTGCCTGCATTTCATGAAATTCCTCATATTTGACGACCGTTTTTCATTTTGTCCCGCTCTGACAGTTTTTTGATGGCCGCAATAGGGTATCGGAGTAAAAACGCCTTCGGTTATCCACAAAAACCCGCAAAGGAATAGCTTCAGAAAGCAGCAAACATAGAACATCAAACCCGCAATATATTCTTAACCCCTGTTCTTTAATCCCCTGCGTTGCTTCGCCGCAGGGAAAATATTTATCTTTGAAACAACTGTGGATAATTACAAAAATGCCTTCGCTTGCAGCGGCTAACGCCGCGCCGCTCAGAATATAAAAGTACCTCCCACCGCTTCGCGGCGGGCATATGGACTTCTGCCTAATGAAAAACAAAGCCCCTCCAACCCTCTTACAAAAAAACGCTGCAAATACTCAGCCACAGCGCCCAACCCATCACTGAAAAGCGCCGCCGCCCCCGCCCGAAGGGCGGGAACAACATCGCTTTCAATAATGGATGTTGTAACTAAGAAATCACATCGCTGTCAGTCTTCTGGCTGGACGTACTGAGTACACGCTCGTAAGCGGCCCTGACGGCCCGCTAACGCGGAGATACGCCCCGACTGCGGGTAAACCCTTGTCGGGACCACTCCGACCGCGCACAGAAGCTCTGTCATGGCTGAAAGCGGGTATAGCTTAGCAGGGCCGGGATGAGTAAGGTGAAATCTATCAATACGTACCGGCTTACGCCGGGCTTCGGCGGTTTTACTCCAGTATCATATGTAACAACGGAGTGCCGCCTTCCATGCCGCTGGCGCGGCATCAAAAAAGAAAGCCCCCCAATGACGGGGGAAATAGCGTTTTGATCAAAATAATAATGCTAGTGTGCTTCTTGCGTGCCATCTTAACTTCCAAAGGGTACTCCCGCCGACATAAGGCGGGAGGGGAATTTGGACGGGCTTGAAAAGCCCGAAAGGTGATCAGCCACGCTGATGTTGAATGTATTGCTTCACAACTTCCAGCGGCGCACCTCCGCAGGAGCCAGCAAAGTATGACCTCGACCAGAGCACGGCTTTTCCGTATGCCCCCCGCAAATCAAGAAATTCATTTCGCAGACGGCGGGATGTCACTGCTTTCAGTGAGTTAACCAGTACCGAAAGTTGCACTGTTGGTGGGTATTCGATCAGCATGTGGACATGATCGACGTCTCCGTTACTTTCTTTAAGTTCTGCCCCAAAATCACGGCAGACTTCTGCTGCATACTGATGGAATGCTGCACAGTGTAACTCTCCCAGTATCTTTCGACGGTATTTTGTTACAAAGACAAGATGAACATGCAAAAGGAAGGCAGCATGTCTTGAACGATTGATTTTATATTTTTGCATTGAGGTTGGCCGTAAAAGCAGTAATATTGTAAGCAAATCTACTACACACTGAATGATGATGCTAATCCTGAAAGCCTACAAATTCAGACTGGAACCAACGCATGAGCAGTCGCAGCGTTTGCGGCAGTTATGTGGTTGCGCCCGTTTTGTCTGGAATTTAGGTCTTGCGGAGACAAAGCGCATACTTGGCTCAGGCGAAAAGCTACCTTCGGCTTTCGAGTTGAACCGGATGATTACGGTGTGGAAAAAAATGCCGGAATACATCTTCTTACAGGATGCTTATACCGACAATCTGCAACAAAAGCTGAAAGACCTGCATACCGCATGGAAACGTTGTTTTGATAAAAAGCTCGCAGCTAAGGCTCCGGTATGGAAACGAAAAAATGAGGGCAGAGACTCAATCCGTTTTGTGAACTTTGAGAAATATTGCTGCCTTGAAAATCGCAGAGTGAAGCTACCGTCAGGTCTTGGGTGGGTAAAATTCCGGCAATCTCAACGTGTGAACGGTAAAATCAAAAATGCGACAATCAGTCAGTTAGCGGGACAGTGGTATATCTCGTTTCAGGTTGAAATTGAAACGGCAGAACCAAATCACACAAGCACAACGATAGTCGGGCTGGATGCTGGCGTGGCTAAACTTGCCACGCTGTCAGATGGCACAGTCTTTGAGCCTGTAAACAGTTTTCAGAAAAACCAGAAGAAGCTGGCGAGACTTCAGCGACAGTTAAGCCGCAAGGTCAAATTCAGCAACAACTGGCAGAAGCAGAAACGCAAAATACAGCGACTGCATTCCCGTATCGCAAATATCCGCAGGGACTACCTTCACAAAGTCACAACGACCGTCAGCAAAAACCACGCAATGATAGTCATTGAGGATTTGAAGGTCAGCAACATGTCAAAGTCAGCAGCGGGTACGGTCAGCCAGCCGGGGCGCAATGTCCGGGCAAAATCAGGTTTAAACCGTTCGATACTGGATCAGGGCTGGTATGAAATGCGCCGCCAGCTTGAGTACAAGCAGCTCTGGCGTGGCGGTCAGGTGCTTGCTGTTCCGCCAGCGTACACAAGCCAGCGTTGCGCGTGCTGTGGTCATACTGCGAAAGAAAATCGCCTGTCACAAAGTAAATTCAGATGCCAGGTATGTGGATATACAGCGAACGCCGATGTAAATGGCGCTCGTAACATTTTAGCGGCGGGGCACGCCGTGCTTGCCTGTGGAGGGATGGTGCAGTCAGGCCGCCCGTTGAAGCAGGAACCAGAGGCTGAGCAATCAGCCGTCACCCACTAATGGGAATCCCCCTCCTGAGTCACGAAGTGCGAAGGTGGGGGAGGATGTCAATCCAGGCACAAACTAAATTTAACGTTTCGTTAACTACAAAGAAATCCGATGAGTTAATCACTTGCGAAATAAAAATCATATAGTAAATTACTATATGATTTTGCGTTGTTTCGGAGAATTAAAAAATGCCTTTGATTGAATACATCAACAAGTTCTATCGGGGGAATCAGGCATCGTTCGCCAGGCTGACCGGAGTTCAACCTGCGCAGGTAACACAGTGGATCAACAAAGGATTCATCGTGGTGAATCACACACTGTATAGTCCACGCCGAAAGCTGGGAATTTAATTCCTGGTCAATATTTGACATCCTCCACGCCCTGAAGGACGGGGATATAAGGCGCACTGTATCCTGTTGTCTGGCAGAAAAAAGAAAGCCCCGTAGTTAATTTTTCATTAACCCACGAGGCGATCCCAATGCTTCAACAACACCAGGATAGACTTTTACCGCCCTTTACGCAATACCATGTATTTTCAAGTTTCTGGTCGATATATAATGTTGTTGGCGCTTGAGGCTTTCTGCCTCATGGCGAAAAGGTGGTTTGTATCTTGTTGTGCGGCAGAAAGAAGAAAGCCCCGTAGTTAATTTTTCATTAACCCACGAGGCGATCCCAATGCTTGAACAACATCAGGATAGCCTCTTACCGCGCGTTACGCAAGGAGAAGAAGGCCATGAAACTACCGGGAAACGCCCTTATCTGGTGCGTATTAATCGTGTGCTGCACGTTGTTAATATTCACACTCCTGACCCGGAATCGCCTGTGCGAAATCCGGCTGAAGGACGGATACAGGGAGGTTACGGCAAGCATGGCTTACGAATCCGGCGGTAAGTAGCAACCCGGAGGCGGGCGAAAGCCCGCCTTTTTGGGGATGATGTGGTTCTGGCATTAAGCGCCTTTACAAAGGGGGTATTCTGTACGGATACCCCCTTTTTCCATGTATTTTCAGGATATTAACTGGTGAATATGGTATAATTAGTCAATATGACAGCAATTATTGAGGCGAGTATGCAGCCCAAAAAAACACCCGTGATTGTGGTCAAAAAACGCCGCGTTCTGGTCATGCCAGAAAATCCTGTGGTGAATGAAAAGCCGCAGGAAGTACAAAAATCAGCAGTTAATGAAAACAAGAAAGTACAGAAGAAAGATGCTGTAGCAGAGAAAACACGTAAAAAGCAGCCTCAGCCCTGGTATTTGAAAAAACAAATCACTTTTCCCCCAAAATATCCGAAAGAATATTTTGAAAAGTGTTTCAATAAAGTTCGTGCTGTTTTTCCTGAACTATGGACAGACGAAAAAAAGAACTTGCCCCTGAAAAGCGGGATTCTCCAGGACGTTGAGAAATACCTGGCGGATAACCCGGATGTGGATCTGACGATTGAAGAGTGGAATTGTGCGGTTCAGGTGATGACGTTCCGGTGGCAATATCTTCAGAATTGTACTGTACCAGGCGCAACACGTTACGACCTTTACGGGAAACCTGCTGGTACAGTAAAAAAAGCACACGCAACTTATGCGCAACTGGTTCTCGATGCCCGTAAGAAAGCCAGCGAGAAGAAACAATTAAAACGTAAGGGATAAAGCCCCGAAGGGCTTTATCATTTCAGCCTGGCTGACTGTAGAATCCAGTGATTTTTTCCGTTTTCGTCCAGGCCGTGTTCGTAAGCTGCAAGAGCCGCTTTCATTATTGCTGTTTTTGTCTGGCCTGTTCGCAGTGCTTCCCGTTCCAGAATATCTTCGTAAACGTCCCCAAGCCGGACATTGGTCACTTTAGTTGCTCCCGATTTTTTGCGGGTAGCTTCATTGATGAATCGGGCTGTGTCTGCATTGGTTTGTTCTGTTTTTTGTCCTGGTATTACCGGAGTTTTCAAGTCAAGAGCCATTATATCACCTTTCGTGTGTTACATTAGCTTTACTGTGTAAAGTTAATGATGTAAAGCTGTCGTATTACATCCATTAGGGATTGATGCCTAACTCTTGCGCCAAAAGCTCTATTTGTGCTTTAGCTGTAGAAAGGCTGGATGCGCGACGTACATCATGAACGCCAGCGCCTTCATTACAGGCCGCTTCAAAAATATCTAAATCTGAAATACGTGTACGAAGTGGCTGTATCCACACCGGGTTCTCCCGGAGTAACTTGTCGAGATCAATCGCTGCTTTTCTGTGGCGTGGTTTTGTCGTGTTAATTCTCGTGAAAAGTACCCAGGGTTGCAGGTCTGGATTGATCTGTTGAGCTGTGCGAACTTTTTCTGTTACATGAGTCAGCGTTTCTGCTTCGAAATCTGAAGACGGCTTAACGAGAGTAAGCAGAATGTCAGCTACAGTTAAGGCACTGCGAAATTCCTTGCTGTCATGCCCTGGACAGTCAATGATCAGCACTTCACATATTTTTTTCAGGCGCTTAATGACTTCTGATACATCGCCATACTCTTCATAAACAGGAACCGGTAATAAGCCATTCTGCTTTCTTTTTTCGTTCCAGCTAAGAATATCATTGTTTTTATCGGTCTTCAGAATGATGACCGTTCGTGAATTATTTACCAGATAAGAAGCCAGGTTTGTTGCTGTGGTGCTTTTTCCTACACCGCCTTTATCTGATGCGACAAGTAATGCTCTCCCCATAGAAACCTCGGATATACAGTGTAAAGGTTTATTTGTCTTTACATTGTATTCATTATAGTTACGTTGTAAAGATGTTTGTGTCTTTACAATGTAATTGTTTTTTGCGCTTTACATTGTGTGTATGTTACAGGGTAACTTTTCAGGATGCGATTTTGCCTGGTGTGTTGTTGCGCCCGTAGCTCGCTGACGCTCACACGCGCAGTACAGCGCCCCGCCCTGCTGCAATGCACCAGGACGGCGTAAACTCGCTGGCGCTCGCGCTAACTGCGGCATTCTGCGGCGCAAATCAGCATGATCATGGCCTGTCAGTCCGTTGGGCTTTGCAGGGTGAAGGGATGGCCCTGTGCATCCTGGTATGTTCGTCAGCGGCTGCGCCGCGCTTCGGGGCGATGTGGCCCCGCTGCTCCCTGGTATTTTCTTTCGCCCCTTCTATCCGCTGACGCGGATTAAGAAATGAGCAAATCATCAAGTAAATTGCTGTTTTCAGGCAATAAATCTTGATAATACATCTAAATAGATGTATTATTATTTCATCGAAAGGGGATTGGCTCCTTTCCTTCTTAAGTCCAAACGGACAGGAGATTTAAAATGAAAACATTGACTTTTAATAACGGCACTGTTTCTGTTGGCGATGTGTTTGTATCTTCCTGGGGATATGAGCAAACGAACGTTAATTTCTACCAGGTTATTTCTGTTCATGGCAAAAAAACCGTAACTGTTCAGGAGATTCGCGCTTCAGTTCATCGGACTCATTCAATGAGCGGATATAAGACCCCATTACTTAATGATTTCTGTGGTGAGCCATTAAAACGGCGAGTGCGTGATTATTACAGTACGCCAGCAATTGAAATTGAGGAATTCGAAACAGCATATAAAGGATCACCGGAAGAAAAACACGAGTTCACATCATACTACTAAGAAAATAGTTGCAGGGGGTATCCCCTGCAACCATGTTAAAAATGTTAATAGTAGTAGTATTTACCCAGGGGGTTTTAGTATGAATGATGGGGTAAAACTCCGTCCTTCAGGGCGTGGAGAATGCCGGGCTAATGCAGCAATGTAATGTTGCGCCCGTAGCTCGCTGACGCTCACACACGCAGTACAGCGCCGCGCTTCGGGGCGATGTGGCCCCGCTGCTCCCTGGTATTTTCTTTCGCCCCTTCTATCCGCTGACGCGGATTAAGAAACGAGCAAATCATCAAGTAAATTGCTGTTTTCAGGAAATAAATCTTGATAATACATCTAAATAGATGTATTATTGTTTCATCGAAAGGGGATTGGCTCCTTTCCTTCTTAAGTCCAAACGGACAGGAGATTTAAAATGAATATCCAGGAAGCATTAAACGTTTTTGGATTATCCGGCGAATTAACTGAAAAAGATATCAAAGCAGCATACAGAAAAGCCGCTTTAAAATATCATCCAGATCGTAACCCGTTAGGGGCTGAACTGATGAAAGCGGTAAATGCAGCTTTTGATGTCTTGATGGCAAATATTGATAAAATAAATCAGTTCCAGAGCGCTGATGAACATGCACGATATAATTACGGTGATGACCTGGAAAAAGTATTAAACGTTCTTTCTGGTTTATCTGGTCTGGTATTTGAAGTGATAGGTAACTGGGTATGGATTAGTGGAGAAACTATTACACATAAGGAAACTTTAAAAGAAATCGGGTGTAAATGGGCGGCAAAGAAAAAACAATGGTTTTATCGTCCAGACGAACATAAAAGTTACTGGAATCGTGAAGAACACACGATAGAAGAAATCCGCGCAAAATACGGTACAACCGGACAGCGCAGGGCGACAGGGTGGCAACGCGTGGAAACCAGAGCGTAACCAGAACGGGGGCGAGAAGCCCCCGATATCATACCAGCCCGTCTTTTGGGGCATCGAACCCAAAAAGACGGGAACAGCAGCCACGTAGCAAAAAGGGAAACAGATGAACTCTTTTTTTGAACAATATCACCCTGTCTTTGAAGTTGTCTGCCGTATCCTGGGGAACGGCTGGCGCGTGAACAAACTTGATGATTGTCCGTCTCGTATAAAACTGACGTCACCGCAGTTTAAAAATTACTCTGTGCATATTCGGATGGAAAAAGACCGATTTTCTGTTGTGGGGAGTGTGGATAGTCGTTCCTGGAGTAGTCCGTATCATGTTTGTACGTTATCCAGGAAGCGGAATCCCGTTGATATAGCAGCCAATATCGAGCGAAAAATTTTGCTAAATGCTTCGCAGGAGGTGTTACAGGCGATTGAGTATGAGAAACGCCAGGCGGCAAAAAAGGACGAAATTCTGATCCTGAAAGGTATGTTATCGCAACTTGTCCAGCTTGAAAGCTGGTATGGGGCATTGACAGGCTTTAAAGCTGAAAATGGATTGAACGGTAAAGTCACCGAGCAGGGCGAGCGTTATGATTTGCAGATTAGGGGTTTGAGTATAGATCAACTTGTTAAAATTACAGGATATTTGAAACAGTTATGAGAAAAAACAAAGGGCGATTGACTTATTATCTTGAAGTGATTGATAAAAAATACCATTTTGTAAAAAAAATAAGCAGTTATTCAAAGGAGTTCACTGACGGAAAAACAAAAAGAACAAAGAGAACGTTAAGTGAGCTGGTTTTTAATGAAAGTGAGGTCGAGGCAATAGACTTTACAAAAAATGGTTTAAGACCTGTTGATAAGAATATTCTCTTAACTATGGTGAAAGAATATAAGGAGAGTGATGCATGACTATTGAAAATACACCGGAAAACATAAAGAAATTACGTAAAAAAATTGGCCTTACCCAGACAGAGTGCGGTGAGATTTTTGGCGTAGGCTTAAGTACATGGCAGAAAAAAGAAGCTAAAACCCACAACCAGCTTAATTTATCGAAAGGTGAATTTGAGTACCTGTTATTACTTGCAGGGGAGCACCCTGAATACGTCCTGCATAAAAAAGCTGAATCCCGCTCACCGGAGGAACCGTGAGCGAGAAGAAAAACACCACGACGCAGAAATCTAAGATATACAGTGTAAAGGTTTATTTGTCTTTACATTGTAATTGTTCTTTGTCTTTACACAGTGTGTGTTTGTAACAGGGCGACTTTACATAATTGCGATTTTGCCTGGTGTGTTGTTGCGCCCGTAGCTCGCTGACGCTCACACGCGCAGTACAGCGCCCCGCCCTGCTGCAATGCACCAGGACGGCGTGAACTCGCTGGCGCTCGCGCTAACTGCGGCATTCTGCGGCGCAAATCAGCATGATCATAGCCTGTTGGTCTGTTCTGGCTTTGCAGGGCGAAGGGATGGCCCTGTGCATCCTGGTATGTTCGTCAGCGGCTGCGCCGCGCTTCGGGGCGATGTGGCCCCGCTGCTCCCTGGTATTTTCTTTCGCCCCTTCTATCCGCTGGCGCGGATAAAAAAATGAGCAAACCATCAAATAACTTGCTGTTTTCAGGCAACAAATCTTGATAATACATCTAAATAGATGTATTATTGTTTCATCGAAAGGGGATTGGCTCCTTTCCTTCTTAAGTCCAAACGGACAGGAGATTTAAAATGAAATTCACTTCAACCACGAATCATGTTTTCACATTCGAACGAGTAACGCTTTGCACGATTGTTCTTATTCATAAGGATACGGGACAACAGTATGTTGTCATATTTACGGATAACAATAAAATTCGTGACTATAAGACCGGAATTGTTTCTCAGTTTGGTGAACTTAAGCAGAGTGATATTGATTTAATTCTTTTCTATCGGGACGAATATGAAAAATATTTTGACTCGTTAAATAATGGCGAGGAGTATTTGAGTTTTAAAGAATATATCGGATGTATAAGGGGAAAATAAATTATGAAAGGTGTGATCAGGTTGCTGGAACAACCTGATCACGTTATCGACAATCAAAACTGAATGAGGTTTTTAAATGTCAACGCGGCAGATCGTACAGCTTGCTGACTCCCTTGTCAAAGGCTGGGAAACCAGAATTCCGGCAATGACGTGCACGGAGTGGGATCAATTCCGGTGGTGGTTAAACTACCTGCAAGGGTATGAAATGTTTTAAATAACCGGGATAAATTCATATATGGGGAGAGTGTCATGCTCTCCCTTTCTTATTGTGTAAGCGAGTTGTATTACATATTTAGCTTTACGTTGTAAAGATTTTATTCGTTGTCTTTACAGTGTGGTGCGTTTTCCGGCGGTTGCGCTGCGCTTAGGGGTATGTGGCTTCGCTGCTCCCTGGTATTTTCTTTCACCCTCCTATTTGTCCGATGACTTTTGACAACAACGCTATTTGTAACTATAATTAGTTGCATGTTGGCAAGGGATGGGATTATAGCGTATGGGGAAAGCAGATAAGCTACTGGCAAAATTTTTAAACAGTAAAAAAACGTTTGAATGGGATGAACTCGTTGTTTTGTTTTCTTCTCTAGGATATGTCAAAAAGGAAATGCAGGGATCAAGAGTGCGATTTTTCAATGCTGAAATTAATCACACAATATTAATGCATCGCCCACATCCAGAAAGTTATATTAAAGGTGGAACGCTGAAAGCTATTAAACAGAACTTGAAAGAGGCTGGGGTTTTATGAAACATTTAAAATATAAAGGATATTTAGGTACGGTTGAGCCGGATTTTGAAAATAATATCCTGTATGGAAAGCTGGCATTTATTCGTGATCTGGTGACTTATGAGGCAGAAACATTGGCTGACCTGGAACGGGAATTTAAAACATCGGTTGATTTGTATTTACAGTCCTGTGTGGAGGACGGAAAGGAGCCTGACACGCCCTTTAAAGGTGTGTTTAACGTCAGGCTTGATCCAGAGCTGCATCGTCGGGTCGCTGAAATGGCGATGGAAGAAGATTTGTCGCTGAATGCCTTTGTCAATAAGGCACTGGAAAAAGAAGTCAGTCATCATCGCGCAGGGGCTTAATCGCCCCTTTTTGTTGCTCGCAGGCGTTGATGTTTCTTGGTATTACGGTGTAGTGTGTTGTATTTACAAACAATGCTTTACGGTGTATTACGATGCATACTCTTTAATTGCTTGCAATCTCTTTTCACGTTCGTTGCTTTGTTCAGCTTTTTTCAATTCACTGAATATGTACTCTTCATCTTTACCCGTGAGTTTTGAACCGGAGAGCAAGCGATTAACAACTTTACGTCGAATTGCAGGGTAATCTTTGGTTTTATCGAGTATGGACAATATGCGGTCACTACTAAGAATCTGGCCTTTCCCTGATTCCACATAATGTAGAAAAAATGCGATTGCGGACACGCCCAAAAGCACTGTAAGGACGGCTACACCATCAAGACGAAACTTTGATATAAAGGAATACGCGACCACTCCAATAATTGCTAAGCAGAGACATAAATAAATTAAGAGGTTTTTGTTGCGTTGTTTTTTTTGTGCGCGATCATGATCATCTATGATTGCCAGTATCTTTTCTGGAGTTGCTTGAGCCATTTTTTTTCCTTATCAGGGAGACGGAGTGTTCAATCATCTGGCTTTACAATGTAAAGCTGTGTTTGTCTTTACGGTGTAAATAATGAGATGTGCAGTTAAAAGTGTCAATCAGGTATCATATAGTAAAACACTATATGATTTTCTATGTATCACATGCATAGCGATAAAACTGGATAAATGATTTACACTGTAAAGATTGAACTGTCTTTACAGTGTGTTTCATAAGTCATACGAATGAAATAATTTGCTTTTGTATGTATGCGTGTGTATAATTGTTTTCAGGTTAGGGAGGACATATGAAATCTGCTGACCTGTTGAAAGAACTGATAGCTGCTGGTTGCGAGTTGAAACGGCATAAGGCAAGCAGTCACCAGATATGGTGGTCGCCAATAACAGGAAAAACTTTTCCTGTTCCTCATCCTAAAAAGGACTTGCCGCTAGGTACTGTCAGATCGATCAAAAAAATGGCGGGGATTTAATCCCCGCCGACTTTGGAGGTAGCCATGTTCTTTTCTGTTGGTGTCGAAACACCGAAAGATGATCATACAGCATACGGTATCACCGTTCCGGTTTTTGATTGCTTTGATTTTGGATGTGTTTCTGCTGCTGATTCTCAGGCAGAAATACCCGCAATGGCGCGTGAAGCGATATTGGCAATCGTGGAAGAGATGGTAATAAGCGGTGCTCATTCTGTTGATGATATTCATGATGAAGGGTGTTTGACTTATTCCGCCAACCCAAATTATAACCACTGTGATAGCTGGTTTGTAATTGATGTTGATTTGTCAGAAATTGAGGGCAAACAACAACGTATTAATATTTCATTGCCTGATGTATTGATTCGTCGTATTGATGGATATGTGAGAGAAAGTGGTGGCGTATACAAAGATCGTAGCCATTTTCTGGCTCAGGCGGCACGTCATGAACTTGCATATAAATAAAACTATCCCCGCGTTGTGCGGGGATTTTTTTTGATAGCTAACCGCTCGCCGCAGTCCAGCGACCGAGCATAGCGAGCGAGCGACGAGGAAGCGGAAGATCCACCGTTGCCAGGAGAAAAGCAGGTACGCACTATGCGTTCTGCCCTAGTGATGCTTTCACAAACCACGCAGCCTGAACTATTACGCCATGAATCCAGCGCAAATCGGCATCACGATTTTCTTTTTTCATTTTTTCGCCACTTAAAGCCTTGCTTACGTGACTTTTTACCAGATTTTCGTGTAATTCTTTTGCTTCTTCAATTGTGAAACCACCAGGCAAATTTGGCGCTTCTGCTTCCAGCGATGCCAGAGAAATTTCATAAGCCCGACGCTCAATATTGTCTCGCACGTCCAGGCTGCCGATTCGCTCCCTGATTTCTTTAATTAGTTTCTTGTCGTCGTCGGTAAAAGTTGTCATTAGTGACTCCTTTTCATATCTATGGTTTTCTTGAATCAATTTCGTCTAGTTTGAGTTTTATATTTTGCTGGTCTGATTTTGGTAATTTTCTCATGCTCTCTTCAGTTATTTTTTTCATATCAAGAAAAGCTCTTGCATAGAAGCGCAGGCTTGCCTCTTTTTCATGTTGTGCAACATACCAGGTTGCGCCAGAAAATGCCGCAGACAGGCATAATGTGCACAAGGCACAAATTGCAATGGCTGTTCTTGTACTGAAGGGTTTTACAGCCTTAGATAATGACTTCAACTTTTCTTCATATAATTTGTTGTTTTTTTCCTGGTTGCGTTTAATTCCAGATATGAGTTCAATAATATCATCTGTTAATTTTTCTTTTACCTCAATACTTGTTTCTTTTAGATCGTTTTTTCCCTCTTCTACTTTTTCTTTTATTTCATTATCCAGATCGCCGCACGTCTCAACAATATTCTTGAATCTTTGAATATAGTCATTTTCTACAGATGAAACGACCTGTCTGAAGCCCTCAGACAGGGCTGTGAAGTTAGCTTGTAACTCATTGCTGAATTTTTCCTGTATCTTTTGGTTCAGAAAAATTAATGAAATAACTGGATCATCTTTTGATATTTTATGCCCTGTCTCCTTGAATACATCAACAATAAAATTATCAATATCTTCACTGGAAACAGCCTTTTCAGGCTGTTTTAATTCTGGCTGATTAACTGTACTCATATTATTCACCGTGTTCATCGTCATAGATGCTATCTAATTGTTTGTAAATGTCATTGAAGACTCGTTTTAATCGTGATTTAGCCATTAATCCAAAATGGTCGGACTCAAGAGCTTCTTTCAGTGTTAGTGATTTTTCTGTTAGCTCTTTGATGTCAGAGGCAAAGGCATCACTTTTTCTGTCCTGAATAACCACAACGCCAGCGATTACATCCTTGTTTTTTTCAATGAATTTAGTTTCTATTAATGGGATATTTTCAAGTGCTGGAATACCCTGAAATTCATTTATCCAGACAATAAGTTTTACCTTACTTCCTTTAACCAGTTCTTTCAGTTCTTCAAATCCCTGCAAGGTATCCGCAAGAGCTTGTCCACCAACGATAACAGTATGGATATAAACGTCTTGTTCTACATCTTCAAACATGTCCATAACACAGTTATCATTGAAATACTGAATTAACGGTAAAAATGTAGATGCGCCATTGTCAATAACAAAAGTATTCTGACTATTGGTCAGCATTGATTCGAACATTGGATCGAATTTGGATTGTATTACTTTGCTGTTTTCAGTGATTTGTATCAAATCTGCATTTAATCTTTTTACTTTAACCGTAGTTGTATTAACCGGGTCTGTGTCACCGACTACGATATTATCCATGTGATTTTCATCAATAAAGTATTGTGCCAGTATTGCGGTCGCGAATGATTTTCCAACACCACCTTTACCCTGAAGGATGAAATTGATTGAGTTTTTCATTTATAATGCTCCTTTACTTAAAGATATTTATTTATGAATTTTTCATTCGTAGCACTTGGATCGTGCTCTATTTCTTTTTTGTTCTCACTAATGTTTGAAAAAAAACCTTTTACCCCCCCTCTCGGTTCATCGCCTTTAGCTTCATTCTGATCAATTACATCTATGGTTTTTGCCTGTTCTTTTGGTTCAGTGCATTTTTGTCTTTTTTTAGCCCTCTCTGTCATATTTATATAAGTCTTTTGGGCTAGTTCTGTCTTGGTGTTGTTGTTTATGTAATCAGCTATTTGCTCCCATGTATAATATTTCTTTGCTTCAAGTATATTTTGCAAATACATATTGAAGACAGCCTGCTTTGATTTCGATTTTGTTTCGTTTATAGCCTCGCATATTGGAGATAATAAACTCTCGATCTTTTTCATTTGTTACATTTCCTCTCTCTCTCTACCTCCTACATTGGCATAATACCTTATTTAAAGCAATTTGCTCGCCGTTTGTGTGGGTGATTATGTGGGTTGTTTTGTGGGTTGTCAATGGGGGTGTGGTCTTTTTTGTGGGTCAAAGTGTGGGTGATTGTGTGGGTTGTGTGTTTTTGGTTGTGGGTTATTATGTGAGTCATTGTGTGGGCTGTATGCACGGCATTTTTTGTTCTTCTAAAACACAGGCACGATACCGATTGTACACAAGACCGTTCCGGTCATGTTACAATCGTGTGCCAAAGGGGGACGCCCCTTTGAAACCCCGAACGATGCAGCCGTTCTTATGCGGTTCTACGAACCTTGAGGACATATGAAGAAAAAAACCAACAAAAACGTTCATGTAACATTCAGACTTACCGAAGAAGAATATGCTCCGTTCGATAGGGCTATAAAAGAGCTTAATATTAGTAAGTCTGAATTTTTCAGGCTGCTTACTATTGGTAAAATAAACACATATGCATCTGATAAACGTAACATACCAGAATACAAACGTTGTCTTTCTCAGTTGAGTTGGGCAGGAAACAACATAAATCAAATAGCGCACCGATTAAATTCAGATCATTTAAAAGGTATTATATCAGAATCGCTTTATAAAAAGGTTTTAAATGGACTAATCGGTATTCGTGATCGCCTTCAGGAGATAGCTAAATGATTGTCAGATACGGTGGTGGTAATGATGGTATCGTTGATTATCTGATAAATGGTCGCAAAGCAGAACGCCAGTACACGCGTGATGAACTTGATCATCGTGTTGTTCTTGATGGTGATTTACAGACTACAGATAAGATTATTGATTCTATTGAAAACAAAAGTCAGGAACGTTATTTGCACATTACTCTGTCTTTTCATGAAAGTCATGTGTCGAATGAAGTGTTAAAGGCTGTTGTTGATGATTATAAAAAATTATTGATGAATGCTTATCATCCTGATGAATACTCTTTTTATGCGGAAGCTCATTTGCCTAAAATTCGTCATATCCAGGATAATAGCACTGGTGAACTTGTTGAAAGAAAACCACATATTCATATTGTAATTCCTAAAGTAAATTTGATTACTGAAAAGTTCCTGAATCCAGTGGGAGATGTTACCAAAGGGCATACAATTGAACAGCTTGATGCTATACAGGAATTTATTAATAATAAATATAATCTTGATAGCCCTAAAGACTATCCGCGTAAAGATGCGGATTACGGAAAAATTATAAGTAGAGTTAAGGGGGATCTTTACAAAGAGCATCACTCTGAATTGAAAGGTGAGTTGCTTTCGCGTATAGAGAACGAGAAAATTGAAAATTACTCTGTATTCAAAGATATTGTTGCAGAGTATGGTGAACTTCGTATAAGAAATGCTGGTAAAACCAATGAGTATCTGGCTGTAAAATTGCCTGGAGATAAAAAATACATAAACTTAAAAAGTCCTCTTTTTCGTCAGAATTACATTGAGACAAGGACATTAACACTTGAGAAACCAACACACAAAGAGATAGAAAAGAGACTTAATACCTGGTTAAATAAAACAAGCCAAGAGATTAAGCATATTTTTAACCAGGCGGAGAAAACAAGGGAACTTTACAAGACATTAAGCCCTTCACAACAAATTGATTTCCTGCAAGAGAGGATAAAAGAATATGACTCAAGAGAAAAACTTAACGAAAGAAATTCTCAGCAAACGTCAGGACGAGCGGGAGGTTACAAGTCGTGTCCTAAAAAGTTTGCCAGAATCCGTCAATCTGAAGCAACAGTCGGATTGTCACGTATGCCCCAACGCGGTATGGTTTACGGAATCAATGGATTCACAAGACCCGACTCTGTCAGTGTATTGTCAGATATTTCGCAGCGTGATCTGGCAGAGCAATTATCGCAAAGAGAACATCCTGGTCAGGATGTGCGACGGGATTATGATAGACAATTCACAGAGTCAGGAATAAAAAGTCTTGAACGCTCTTCTTTTTTGTGTGAAACGATGTTTCAGACTCTGAATGAGGCGGCAGAGAAAAACGAAATTGCAACGATGGCAGAAATTCGCAGAAATATTGATCCTGTCAGGTTTCTGTCTTCTGCCGCCGAGCGTTTTAATATTATTCCAGCGCAGCATAAAATCAGAACAGCAAAGGACGGTTCCCCCCGGTTTTCTGTGGGGAATCGTAATATGAACGCATCTGATTTTCTGACAAAGCATATTAATCTTGCCTGGAAAGATGCGAAATCCTTTCTGCTTGAAGTTTATTCACAGCAGTTAGAAAACACGCCATATACGCGCTATCCAACCTACAGACGCCTTACGCATCATGAAGCCCGTGAGCGTCTTAACTCACTGAATTTATCGGAAAAAACATTACGAAATACTATCAGGTTTGAGCGTGGCAAGCTGTATAATGATCTGCGAGAGATGAGACGCGAGTTGAAGTTAATACCACGTGAACAGCGTGATATTGCCGTGGGTGTGATTGTTTACAAAAAACTAACCACACTTGAGCGTCTTTCTGAACTCGACACAGAGGGGCGACACATAATTCGTCAATATCATGCTGACTGGCATAAGGATAAAGATGAAATGAAAGCCCTTGAACGTCTCAAAAGCTATCTCAACTTCGATGAAATCAACGCCATTTCTGCTGACGAACCCGAACTTTCGCTTCAGAAAGCGGTGGATTCCCAGCGCCGCTTAGAAGAGGCGAAAAAGGTTAACTCCAAACTAAAAGATCTTGTGATGGATAAGCAGGATTCCAGGATTGTTTATCGCGATCAGGAATCGGAAAAGCCTGTCTTTACCGACAAAGGGAACTTTGTCGTTGCGGGTAAAAATCCTTCAAAAGAAGAGATCGGAATAATGCTTGAGTATTCCAGGGAAAAGTTTGGTGGTGTACTCAAACTTACCGGCTCTGAAGATTTCAAAAAAATGTGCGCTGAAGTTGCCGCAGAGCAGGACATGAAGATTATTTTACGCCCGGAGCAGTATCAACAGATGATGCTGGAATTAAAAGCAGAACTTCAGGGTAATAAGTTTGAGCAGGTGGAAACACAGGAAAATAGCCAGGAGTCCGAATCCAGAATAGAGAAAGGTGATGCATTGAAGGAACAGGCCACCGAACAGGAACAGGCTACAGAGCAGGCACAGGCTACAGAGCAGGCACAGGCTACAGAACAGGCACAGGCTACAGAGCAGGCACAGGCTACAGAGCAGGAACAGGCCACCGAACAGGAACAGGTCACCGAACAGGAACAGGCTACCGAACAGGAACAGGCTACCGAACAGGCACAGGCCACAGAGCAGGCACAGGCCACAGAGCAAGCACAGGTCGCAGCGCAGGCCACATCTTCATATGATCCTGGTGTTATTACCAGGGCAAACACCCTGGATTCACAGATGCTTAGTAAAGGCACAAATGGTGAATTTGGCTATTTGAAATCTCTGGATAGCGATGAAAATGAGATTTGGGAAGTGCTGGGACATGTACCTGGAGACAGCGATGATATTTTTGACGTTGCCAGTTTTGATAATGAAAACGACGCAAAAGAATTTTGCAAAATTGTGAATGAGTTAGGTATTGACAGAACACAGGCTCTGATTCAGGAGCAGCTTACTACTCAACACGATCAGGCTACTGCGCAGGTGCATAATAAACAGGAAATTTATTGTATAAACTTTAGTCGCTTCCATGATTTGAATGAAGGTATCGTCTTTCATTCTAAAGATGCGGCTATTCAGTGTTATGAGGAAAGCAAATCTTCTGCAATAGAAAAATATGAAAGCAATTATTTAAATGGTGATGGTTTTGATACTGTTGTGCTTATGTCTAAAACTGTCTCAACCGATGAATTATCCTCTTATCCAGAGGGGGCATTAGTAACGTTTGATAGGCCATTTGAAATAATTGCCAATTCATATGAAGAATACCGTACTCCAGTGTATGCGGTTTCTTTTTCGAAGGATGAGTTTAGTGAAGATATTAAGACTTTTGAGTCATTGCACGACGCATCTGAATATAAAAATAAGATGCTTCAGGAGCACGGGCTTAACCAGGATGATATCTTAATCACACCTGTAACAAGAGAAGAAATCGCCTTTAAAGGAATTAAGGACGCTGTTAATGACGCTAATATGGCAGTGATGGAACAGGCGGGGGATTCGTCCAGAGAATCACCAGAAGAGATACTCGCCAGTATTTCAGCTAACGAGCATATGATCTCCGGGCTTGAAAACTTCCTGGTTAAAGACCGTTCTCAGTTCAGTTCCTGCAATGGGGATATCGTTGTGGAGGCGGAAATTACCAGGGGGGAAGGTGGTCTTTATCACCTTGCGGTCGCTGGTAAGCATGGCTTAGAGCGTGGTGATGCAGTTGCCAGAGTGGATGTTACGGAGCAGCAATTTGCCGCGATCACTGGAAAAACACCTTCTGAAGTTTTAACGGGGGACCAAACATCGGCTCGTGTTCCGGTAATTACAGGTATCCATTTCAGTACAAGGGCTATTGAAAATGTGAATAAACTGGAACAACAGAAGGATTATGTCTATTTCTCAACACATGAAGGCCTGAATGCTGAAATAAAAGATTTCAGCTCTCTCAAGGATGCTATTGAGTGGGGCCGGGTTGAATGCGAGCTACATGATCTCAATAAGCGCGATACTGTCATTTATCGCGTCGAGTCTGAACATATTTCTCAGGGGATTGACGCTGTAATGAAAAATGCTGAACGCGTGGAGCGTCATGAAATTGAGAAAGCACAGGGACGGGATTGCACGCCGGAAGACGGCAAGATTCTTGAGGCAATTGATCGCTTTGAGGACAAGTTCAGAGGGGAAGGACTGAAGTTCGAAAGGGAGAAGGCCGAATCCGATTTGCTTAATCACGGATTCACTCGTGAAATGGCAGAAGATGCTCTTGGAAAACAATTTGTCCAGGCCAGAGAAGAACATCTGGAATTGCAGCAACAGCGTGACAATTCACAGGACATGCACTAATTAAGAAAAAAGCCCGGTTTTCGGGCTTTTTAAGGTTGGAAAAACACCTTATGAAGTTTACTTCAAAAAATCGTTGCACTTGGTTTGACAATTCAAGATACAAATTATAAATACTCTCAAGTGTATATTCAGTATGGGATTGCGCAATGATTGCCTAATAAAATTTCTGAAATATTTCTGTATCGCATAATTTTTTATATCAGATAAATTGTACTGGATTTCTTAAAAAATTGCAGTATAATTGCCGCAATTATCCCACCGTTTATTTTTTGAGTAGTTTCTCATGATGCAGCATACTTCTGTGTGGTACCGACGCTCGGTCAGTCCGTTTGTTCTTGTGGCGAGTGTTGCCGTTTTCTTGACCGCGACCGCCAATCTTACCTTTTTTGATAAAATCAGCCAAACCTATCCCATCGCGGACAATCTCGGCTTTGTGCTGACGATCGCTGTCGTGCTCTTTGGCGCGATGCTACTGATCACCACGCTGTTATCATCGTATCGCTATGTGCTAAAGCCTGTGTTGATTTTGCTATTAATCATGGGCGCGGTGACCAGTTATTTTACTGACACTTATGGCACGGTCTATGATACGACCATGCTCCAAAATGCCCTACAGACCGACCAAGCCGAGACCAAGGATCTATTAAACGCAGCGTTTATCATGCGTATCATTGGTTTGGGTGTGCTACCAAGTTTGCTTGTGGCTTTTGTTAAGGTGGATTATCCGACTTGGGGCAAGGGTTTGATGCGCCGATTGGGCTTGATCGTGGCAAGTCTTGCGCTGATTTTACTGCCTGTGGTGGCGTTCAGCAGTCATTATGCCAGTTTCTTTCGCGTGCATAAGCCGCTGCGTAGCTATGTCAATCCGATCATGCCAATCTACTCGGTGGGTAAGCTTGCCAGTATTGAGTATAAAAAAGCCAGTGCGCCAAAAGATACCATTTATCACGCCAAAGACGCGGTACAAGCAACCAAGCCTGATATGCGTAAGCCACGCCTAGTGGTGTTCGTCGTCGGTGAGACGGCACGCGCCGATCATGTCAGCTTCAATGGCTATGAGCGCGATACTTTCCCACAGCTTGCCAAGATCGATGGCGTGACCAATTTTAGCAATGTCACATCGTGCGGCACATCGACGGCGTATTCTGTGCCGTGTATGTTCAGCTATCTGGGCGCGGATGAGTATGATGTCGATACCGCCAAATACCAAGAAAATGTGCTGGATACGCTGGATCGCTTGGGCGTAAGTATCTTGTGGCGTGATAATAATTCGGACTCAAAAGGCGTGATGGATAAGCTGCCAAAAGCGCAATTTGCCAATTATAAATCCGCGACCAACAACGCCATCTGCAACACCAATCCTTATAACGAATGCCGCGATGTCGGTATGCTCGTTGGCTTAGATGACTTTGTCGCTGCCAATAACGGCAAAGATATGCTGATCATGCTGCACCAAATGGGCAATCACGGGCCTGCGTATTTTAAGCGATATGATGAAAAGTTTGCCAAATTCACGCCAGTGTGTGAAGGTAATGAGCTTGCCAAGTGCGAACATCAGTCCTTGATCAATGCTTATGACAATGCCTTGCTTGCCACCGATGATTTCATCGCTCAAAGTATCCAGTGGCTGCAGACGCACAGCAATGCCTATGATGTCTCAATGCTGTATGTCAGCGATCATGGCGAAAGTCTGGGTGAGAACGGTGTCTATCTACATGGTATGCCAAATGCCTTTGCACCAAAAGAACAGCGCAGTGTGCCTGCATTTTTCTGGACGGATAAGCAAACTGGCATCACGCCAATGGCAACCGATACCGTCCTGACCCATGACGCGATCACGCCGACATTATTAAAGCTGTTTGATGTCACCGCGGACAAAGTCAAAGACCGCACCGCATTCATCCGCTGATTTCTCCCTGTATTTTTTCCAAACCCACCGCACACTCCATTCGTATTATGGGCGGTGGGGTGGGGTTTGTTATGCCGTATTTATCAAATAAACGCCTACTTGCTGAGATGAGTATCGCTCTTGTCATGGCGATCGTTGCCACGCTGACCCTTGAGCACAGTCAGATTGATCTGATGGTCGCTGATTGGTTTTATCTGGGTATGGGGCATTGGATGGTTGCCAAGCAAGCTTTTTTGCCAGATTTGCTACTGTATTCTGGACTAAAAAAGCTGCTGATGGCGATGCTGATCTACTTGCTGGTTGCGACCATTTGCCGTGCTTATCATGAGAAAAAGGGCAATGCTATCACTGCCAAGTGGCTTGTCCCAGTGACAAAATTTCGCGTGCGTGAGCTTGCGTATCTGGTGCTGACTTTGATCCTAGTGCCGACAGTTGTCGCGTCATTGAAGGCATATACTCATGTGGTCTGCCCTGTGCATTTGACGATTTTTGATGGTACGCTGCCGTATTTGCCGATGCTTGATAGTATGCGTAACACCATTCCTGATAAGTGCTTTCCTGCGGCGCATGCCAGTAGCGGATTTGCGCTGTTTGCCTTTGCGTTTGCGCCAAGTTTGCGCCGCCGTCGTGGTGCGATCATCATCGTGGTGATGGCATTGGGCTGGGCGATGGGCTGCTATAAGATGATTATTGGCGATCATTTTTTGAGCCATACGGTGGTGTCGATGATGCTTGCGTGGGCGATGTCGGCAGGGCTTGCGTGGGTGTTTTTTAAGAAGGGTGAACAAGTTCAAGGAAAAAAAATCAACCCCAAACAGAAATAACAGCTACAAAAATGGACAGTAATAATGCAATAAAAGTAATGTTGAGCCATATGTTGGACGAGTCTTTTAAGACTAATGAGGCTGTTAATGCCATTGTAATGAAACTAATGATAATAATGGCTGCAAATAAATATTGTACAATGTTTTTTATTGTACTTGGTTGCTCCCCAATTCTTTCTCTGTAATTTGCATCAACATTAGATATCCATTGAAGTAATAGAGCAATAAAACAGCCTACTCCGCAAACGGTGAATTGCACAGACGTCGGGTTTGAATCAAACAACGAGATCGCTAAGTTATATAAGTATACCGCGCCTGAAATCACCCCGACAACTAAAATAGAGATGAGTATAATCCGAAGAACTAATGTATTGCTTTCCTTTAAGGAATATATTAAAGCCGCTTTTTCGTATTCTTTCATGATTTAATCCAGTCTATAAGTATAAGAACCAACAAAACTGTCCAGGATTCATTTTCTGTGAGCATATGAAATCCCATCCAGAGTGATGATACTGGCAGAAACACGCCCACAGCCGGAATTATTAGTCTGGCGGAGAATATCCTTATCAGGAGTATCAATAGTGATAAGAACAGAAAAAGAGTGATCATGTGGAAAAACATTCACATCACCTATAACACTATTAAGATAGCCTGCCAGATATCCATTTCAGACAGGCTATATACAGGGTTCAATTCATTCTATGAAAGGTGTATAGGGATTGCTGATGTTTGTGTCGATCAAAAATCTAATATTAATAGATGAGAACTAATATACATATTTCCAGACGGATGGGGGGATTTTATCGTAGAGTTTATTCATTGTGAGTTCTGCAAGCCTGTGATCGGCGGCGGCATTAAACGACTCAAGCTCAGAAGGAGGGAGGCTGTTTTGGTTTTTTTCAATAATCTTTTCAAGTGTGTCGAGTGTTGAGCAACGTCTTAACTGAAAGAGCCAGTCTTGTTTTGTTTTCATTGTACAATTCCTGAAGAACATCCTTGTCCTATAGTAGCTACAACGATGTTTGATTTCTACTGCTGGATTTATTTCTTACTTTTTTGTGTAAACTCAAATCCCAGTTTCCCGGTTGCTTTGTCAACCAGTTTTACTTTTGCTGAAAATTCTTTGTTTGTTTTTTGACTAATGAAACCTTTCAATTCACTTGTAACTCCTTTTGTTATTAACGTTTCAATTTGTTTATCGGTTAATGATTTGCCGCAGAAATCTTTCCATATCTTAAATTCACATCCTGTACAGGAATAGCTTTTTGGCCTGATAACAATCTGTTTACCACAGGAAGGACAAGGAGAGGACAGGCGTTGTGACTGACCAGACGGCGCGCTGGGGGAAACTTTTATTTCAGCACTGTTCGCATTACTGATCATCGGTATCAGATCATTGTACAGTTCGTCAACGAACTGTTCGATGGTCATTTCTCCGTTTTCAATGAGTGTCTGTTTTTCCGCCCATAATGCCGTCATATCTGGATTAACAGCTATATCCGGCAGAGCATCAATAAGCGCATACCCGGTATCTGTCGGAATAAGTTTTCCTTTTTCCAGGGTGATATAGTTCCGTTTTTTCAGTGTTTCCAATATGGAAGCCCGCGTAGCTGGCGTACCAATACCGCCATGTTCGTCTTTTTTATCCCGATCTTTATCCTTCAGTAACTTTTTAATCACTGGATCAGTGACAAAATCTGCGACACGAACAAGCGCGGCAAGTAAAGTGGCTTCGGTGAATAATGGCGGTGGCGTTGTTTTCTTCTCATTCACAACCACTTCTTTTGTCGTCACTGTTTCTCCTGTGCGAATTTTACAGAGCAGATCAAAAGCGGAATCATCATTTTCGGCTTCTGATTCCTCATCCCCGGTATTTTCCTCACCAAGAAACGCTTCAAATCCGCTGTCTGTTGTTTTCCTGGCACGGGCATAAAACGACTCATCACCACACTGAATGGCAACGGATACTTCCTGGTATGTTTTTTCAGGCATGAACTGAACAAGATAGTGTTGTGCGATTGCCAGGTAAACATTGCGCTCGTCACTGCTCAGCGCGTTTACATCCGGTACGTTAACGGTCGGGATTATCGCCGTATGCGCAGTCACTTTCGCACTGTTAAACGCCTTGCTTTTACGTGTCGAATCAACGTCCAGTGGCTGATCGAATACTGACTTCAGGGCATCGAGAACCTGTGGTGCTTCGCTGAATTGTTCATCAGAAAGATATGAGCAATCTGAACGGTTGTAAGTAATTGCTTTGTATTTTTCACGTAGCTGCTGTGTGATATCCAGCGTTTGTTGTGCCGTCATTTTGAACTTCTTGTTCATGTACTGCTGAAGCCGGACCAGGTTAAAGGGCAATGGCGCAGCCGTTTTTTTATCGTCTGTTGCTGCTGCTTCAACTGTCGCTGGTTTTCCTGCAAGGGAAGCCGCTGCTCCATTCGCCCACGCCTTATCAAGTAATTTACGGTCTGTCAGTGGTGCGAACTCACCAGGTTTCCAGTTCGCCCTGATAACATCAACGCCGCGTTGAAAATGCCCGGTCATGGTGTAGTAGAAACTGGATTTATGATTCTTGTTGGCACGGGTGCGATTTACTATCAGGCCAAGAATAGGCGTCTGAACACGCCCGACCGAAAGTGTCCCTTTATAGCCTTTAGCCCGTGCCGGGATCGTATAAGCACGCGTCATGGATAAACCGTATATCGCGTCGGCTACAGAACGCGCCAGAGCCTTGAGATAGAGTCCTCTGAAGTCACGGTTATTTTTGGGATTTGCCAGTGCCTTTTTCACTGCCGGAAGGGTGTTGTCGTTGATCAGAACGCGCTTAACGGGTTTTGTGTTGCCTGCATATTCCAGGACCTCATCGACAAGTAGCTGTCCTTCGTCATCTGGATCGCCAGCATGAATAATTTCGGTCACATCGGCACGCCTGATAAGCTCAATAATCGTTTTAACTTGTTTTGCGGCGCTTTCTACTGGCTGGTATTTTACGGGATAAAGACGCAAAGGGAGTGTTTCAATCTTCCACTCTTTGTATTCAGGATTATAGTTTTCGGGGGGCTGTGATTCGATAATATGACCAAAACAGTTAGTCACAATAGTATTATCACTTTCAAACCAGCCATCATGACGGGTAAAATTGCCACCAAGCGCCTTAACAATATCATTCGCTACTGCGGGTTTTTCTGCGATAAAAAGTTTCATTTTGTTATCCTTTACATTGAATTAATCTTTTGGCACAACGGATAGCACGTTCTTCTGATACTGCCTGTTGCCAGTTATCTTCACTTACTGGATGTCTGAATCTGCGTTCACAGCCATTAAAAGCAAAAATCCCCTCACGGTTAAGTATATTAGTTGCAATATCCTGGTCTTTAATTCTCAAAAAGGCTGTTACGGCAAAACCAGAATACGAAGAAATTTTTGAAATGATGCATTCATCAGCTTTTATTAACGCGGCAAAAGGAACTATCTTTTGATGACCACACGAAATCAGAGGGAGTGTGTGAATATCATAATAAGCTATTGAATGACAATCCATGCGGGTATCATCAATAGCTGCCAGCCAGAATCCTGTCTGGTCTTTTATTTTTCTGCTAATATCTGAATCTGAAACACCAGGATTTAATAAATATCCACCATAACAGACATGGAAGGGGTGAGCAGATTCATCAATATCAGGAAGTTCTTTTACCGGATTTTCTTTCCAGTCCTTCAGAACACCGAATTCAAGAGCATCCAGCAAAAACTTTCGTTCAAATCCATCAAGCAATGCATATTGCATGTTAAACCTCTCAAAGAAGCTCTGATTTGTTGACCTCATGGTGAACCAGTTCACCATTGCGTAAGACAAACAGGTAAGAAAACTTTCTTTCCTTATCTTTCAGTCAGTTTTTGTATTCAGAAAGAATAAACACCCAGTCATCGGTGCTGTAATGAGATGCGATGACTCATTATCTTTTTAGTGATGAGTTGATTATTTTTAAAATATGCCTGCCTTTAATTAGCATGTTTCTGGAAACAGCCTGATTACGAATACCTCTCATCTGGTCAAACTGATAGGTTAAATCATGTTCTTTTCGTGAAAGTCCAAGAGTGGCTCTTTCATTATCCATCAGGACTGAAATTGCAATATATTCTCCAGATGCACACCAGTCTGCATAACTGGCAACGCAATGTTCCATCTCCTGACCTTCCTGACACAGAGCATCAAATGTAATTAATTCATTTACACCGTTATCAACTTTCTGCCAGTTTATCCCAGTTCCTTTCCATTTTGGGATTACGGGAATAACGTTATTCTTCACTTGTCGCGTCCATTCATCAGATAGTCTCCAGAAAGAAGGCCACTCCTGGTTTTTATGAATTAACCGTTCTTCTGCTAACAACCAGTCAATTGCATGACACAACTGATTCGTTTCCATTTCCCAGCGTGATGTTTGCCTGGATTCAGTATTACGGTCATAAAAGCCAATGTTTTTAAATAAATCACGATGATATTCTAGCCATTTTGAGCATATACGATAAATATCATTTTCTTTTTCTGAGTAAGCTCGAATGCTAATATAATCGATAATCCAGTAAATTGCTCTCACAGGATAGTTCTTGATAAGTGGATGGCGTAATAATCTGACTGTAAATCTGAATGATGCATTATCATGTTTATCATTTAAAGCATATACTAATGACAAACTAACATGTCGTAAACTTCTGAACTCACCATAACTAAGTTTGCAGGGAAAACTGATTTTTTCATATAACTCTTTTGTCTTTCCAGATAGTATAAACTGACCATTTTTATGTTGTTGTCTTAGACGACCAACCATAGGCCACAATCCGAAAAAATCCATATCATTCACAATATGGATTCGCTTTGAAAAATCCAGATAGTCCTCAAATAATGCCTGACGAATTGAATTGTAAGCTATCGAACAATGTGAAGCTCTGGCATATTGTGCAAATATCTTTTTATCTACCATTATCCATATAGCTTTACTCAATGCTTTTGAAGCATTAAATTCATTATCCACCCCTATAATTTTTAATGCTCTCTGATAAATTTTGTTTTTTATCTCTGCGAGTAAAATATGTCTGCTGATATATCGTGTATTATAGGGGTAGTTGAATGATGCTATTTTACCAAGGCAATGAACAATGTCATGTATTCCATACTCATATGAGTAATATACATTGGATGGATATAGTGTGGAAATATAACGACTCTCACTGTTTTCCTCTTCCTCTCCAATGCGTAACAAATAGAGTTTGTCTTCAATTGAATATCCAGCCTCAATCAATGATTTGATGATGTAGCTAATCGCATATTTTAACCAGTCGTCCATGTCGTATGCAGATTTTTTAGACCAGAATAAAAAAATCTTACCAGGAAATGAATTTCTTCTGGTCCATACCCCAGTGTGCTTATCTCTTAGCTTGTATCCCCACAAAGTGATTTTGTTATTCTTTTTGTTTTTTCTAATACGAAAAACGCCACAAATGATAATGGAGATGTACTTTTCATTCTCCTCTGTTTTTACAAGACGAAGCCCCCCTGTATTTTGATAGATTTTAAAAACATCATGCAACGTAAGAATATTATTGTCCCTTGTCTGTTGATTAATTACATACATGCCGCCCCCTGTTAAAAAGAAGCCTTTAATATTGCACAAAGATAAAAAGACAGAATCATGGCAAACCAAAAGAAGAATGCATTTTCTTGCCCCCTTAATTTCTGATACGCCATTCCCACGGTGGAACCGGATTTGCATCGGCCCACAAACCACGTTTTTGTTCCTGCGCTTCCCGTTGCAGAGCCGGTAATGACTCATCCACGTTGTAACGTTCATATACCCATGCAGCGCCAGACTGAACCATAAAGCGGCTGGCATCCGTGCCGTTCGTCGTGAACACATGACCGATAATGCGTCCGTAGCGGTCTTTATGTGAATAAGAGACTGTCACCTGTTTACCTGCAACCAGAGTTTTAAGCTGACTAGTTGACCAGCGTCCAAAAGGTTGTTTCTTTTCAGGGGCATCGATGTTTATCAATCGGACTCGGATCGGAACTTCATACACAACAATTTTTGCGGGTAGTGTTTTAATTTCTATGGTGTCGCCATCGAGAACGCGAATAACTTTCCCCTGAAGAATTTTCTGGTTAAATGTATAAGGTATTCCACCTGCATTTGCAGACTGAAAAGCGGTAAGCAGATAAATAAATATTGTTAATAAGATTTTCATAAAAGATTAACGTTAATTTGTCTGTGCAATGGATAGAGCGTTATTCTTATTTTCTTCCTGCCGCTTCATCGAAAAATGATGATTTTCCTACTGCTGCGGCATCATACTGAAATGAATATCCAAAATCATCGTCATGCTCAAAACCTGTATTTTCTCCATTTTTTAGTCTGGAAAGAATAATCTCAAGTTGACTGATTATATCTTCCCTTTCACTATGACGAGTGCCTTGTAAGAGAATAGTTACTTGTTGCAAAGGATATGCATGTTCAATCCATGCCTGGCTAGGTCCAGAGTGCGTTGTATATAAATTTTTTTGTGCCGAAATATTATTCAGAAAACAGTTTAAGTCATCATTGGAATAAATTTCTTTAATATTGTTGATGTTGGATGTTAGTTTTCCAAGTTGGAATGGTGGAGAATTTTTGTCTTCATTGTTGTCTCCTTTATCGTATTCTGGAAACACAATTAACAAAGGTGCACCATTAGTTCTCTTCATAAAACGGGTTACTAGCCGCCCAATATATTTATTGGACATATGCTGTTCAATGTCCAGGACAGCATATTCCTCCGCTGGGAACATTTTGTTTTTTTCGATGGATTTAATTTTTCTGGTAAGATGAATGTTTCTGATAAGCAAACCTACTATCAGGAAGTAAATAAATATCCAGAATAAAAAATCACTCTGTAGTTGTTCTATCATAAGTGAATCCTCTAATTTGTTAATGGACAAATCATTACCCGTTCATCAATGTTTGTAGGAAGTTCGCCAGGAGTAGGGACGCGTTCTCCTCGCTGGGCGGCTTGTGCCAGTGTTCGTAGCAGCAACACGCTGGCATGAAATATGGCCTGTTCTTTTGTGGCTGCGCTTGTTTCCAGGTCGAAATCACTCAGAGTCACAACGACTTGCCCGTTTCTGTTCTCGACCGTTGCCGGGTAGGGAACAAAATAGGGAGCGATTTTGCTGGTTTGCTTGCGTCGGGCATCGAGTTTACTGACAAGTTGATAAGCCCGAAGGTGTGTGTATCGTTTCAACATGTTCAGGGAGCGATGGCCTGAAATAGCGGCAACCTCAATGACGTTCAATGTACCAAGTTCGAAAAATCGGCTGATAGCCTCATGACGGAGGTCATGAAAATGCAAGTTTTCTATCTTCAGTTCCTGAAGTGCTGTTCGCCAGGCACTTTTGAATCCCGAAGAGGTATAAGAAAAAATATTACCGTTAAGCTGTGTCGGCAGCATTTGCAGGTAATTACGGGCTTTCCTGGATAGCGGAACATCGCGTGGAGCACCGTTTTTGGTTGTTGGAAGATGAGCTACCCCATGTTGCAGATCGACGTGTTCCCAACGCAATGACAAGATTTCACCTTGTCGCATTGCTGTTTCCAGCGCCAGATGAAAAATGACGTAAAGAGCCTGATTTTTTTCTTTGAAATACCGCGAAAGTCGGCGTTCTTCTCCTGAAGTCAGGCGACGATCGCGCCCGCTACTGATTTTGGGTTTTCTGACCAGTTCGACAGGATTCATGCGGCATGTTCCCCATTCCACACGGGCGATATTGAATAATGATGACAACAGGGCAAGTTCAAGACGGACGGTGTTTCCGGTTATTTGACGCCCCGTTCGTGGGTTAATTTGCGCCAACCGTTGATCCCGATAATTGGCAATATCAACAGTGGTAATGTCATCCATATAGCGTTCAGCAATAGGGTGACGTTTAATCACATTTACGCGATAAAACTCCTGTAGATGGCCTTTTTTGTGGATGGATACTGTCTTCAGGTATTTGTCCAGCGCCCGGCTGATTGTCATTTTGCGAATTTTGATTTTCTGAAACATATCCCCTCCAGACAAAAAAGGGGGGAAGGTAGAGATTCTTGCTTCGAAATGCAATATCACCGTGCCAATCCGGTGTGTGGAGGGCATTAGGTGGAAAGCTCAAGGTTACTCAACTTTCCAGCACTGGTTACTTGGGGCAATTTGACTTTTGTGCCATTGCCAGAATGGGAAATGCTGAGGATTCTCATTACTGCCAGGTAGTCGAGAGTCCATCTGGTTCACGAAAATGGTACAAATACGAGCACAAGACAGGTTGTATTGCGTCCTGTGTAACTCTCAATTAAGTTTGGTATCCAAATACTGAAAATACCCCTGAGCCACAAGAGTAGTTTTGGGCCGGATAAGAGGTGATCTGATAAGTTGCGCCAGCAGGGACAGCAAAAGAGATGAATGCTGTCTTCCCATAAGATGGGTTATTACTCGCATTTGTGCTAATCAATGCCCCTGCAACGTATCCCTGCAATCTGGATGTGTTAGCACAATCTCCCCCGGCGGAACCACCATTCCCTCCAGAGGCGTAAACAAACAGCGTACCACTTCCCGTATTGCGACCAGTAAATGATCCTCGGTGTGATCCAAGATTACTGTAACTACCGTTAGAGGAACCGGAAGTTCTCCACACACCGGATTGGCACTGGCCTCCGTGCCAATCCGGTACGTGGAAAAAAATTGGCGCAGGTGATAGCCAAATTGTTACCGCTTCAGCTACTGCGTGGCGTTGGCCTGGAGCAACGGCAACATGTCCATCTGGCAAGAAAGTCATTGGTGGAGGCGGTCAATGCAGATCAAATACCGGATTTATATGGCTCACACGTTCCATGCCTTCAGGCAACAATGCATGGACTGCCAGTTGCGACACGACCGAAGATCAAAATGGTTCAATTACAGTTTACGCCATTTGCCAGTGACGATACTTATCACTGGCAAACCACGATGTAACTACATTTCCCCGACAGGTCAGTTCGGTGAGTACCGCCATAAAAGTTGTATTCGTCATAGAAGCCGCATTTCACAATTCGATTGGCATCATAGGTTGCAACTGACCCCGCAAAATAATTCGGCTTCCACCCGTGCGGATTTTCTGCATTACTGACACTCCAGGTACACTGGCTGGCAGAAAAACCAGATGGCAATGGGATCTGTTGTCCATTTGCTATTTTTCCTGTCAGAACAGTGCTACCTGATGCCCTTCTCCACGTACCGGATTGGCACGGAGGCCAGTGCCAATCCGGTCGGTGGTCTGGTGGCAATAAAGTGAACTACTCCGCCTGTAAGTGGTATCAATCATCTGTAGCAATGAATCACTTCATTGGCGGGAAAAGTGGAGGATCTATCTACTACAAACCCATTCAATGCCCGACCGGATTTATCATGACAGGTACACGCATGTATGGCATTGGTGATGGCGTTGATGAGGAACACGTTGATGCTTATTGTTGCCCCTTCGGTTAAATCCCAGCACCAGGAAGATTCAGGCATGTAACAGTAATTGTGCCGCCGGACTCATAATTTCCCCACGCATAATTATTCACATACCAAACCTTGTTTTGGTCGGAGTATACCTGCTGAAACCCACCAAAAGGCGCACCATTAAGGTATGTCCACGAACAGTACGCATGTACGCCAATGCTCAGATTTCGGGTGTTTTTCCCGATATTGTACGTACTGGTAGTGAAGTTCACTTTTGCCGTTGTCCAGATACCGGATTGGCACGAAAGTATCGCCCCTGTACTGTCGCGGCCTACAAGCCCGTTAGGGGAGCAGGAAGCGCCAGCCGTTGCTGTTTTTTCAAGCTGTAAATACTCGCCTGTATAGAGGCGTCCATCAGCGCGTACAGTTCCACCTTTTACCTGGCCGCCAGTATAAATCCCTTTATTATTCACGCTGCGCAACCAGGACGAATCGGACATATACCAGCCGCCACCATAGGTACTGTTCATCCAGCCTTTGCTGTTTTTTGTGACCAGCCAGCCATTATTCGAAGTGATATCGCCGTTGGTTGTGGCTGTATTTCCTGAAATTGCGCCAGAGAACGTACCGTTACGTGCGCTGATATCGCCGGAGAGCGCGGCGGCGTTGCCGTTTAAAGTGCCGACGTTGTTCAGATTGTTGCTGTTCATATCAATCGCCGTATGCATCCTGTTAAGGTCGGGACGATTGGTTATGGAGTAACGATATAAACGGTCGCTTTCCTGCAAATCAGCGCCAACAAGTGCCATTGCAATATGCCCGGTCGCGCAGTTCAGCCCGTAATTTGAAGGCGTGTCAGACCAGCCGCCACCCGCGCCAATCGCCTGCTTGCTGCTGTTGATGTAGCCGCCAAGCCCTTCAATCATGCTTGCTACCGAGCGCATTCCGGCTTCTGACAGGGACTGACCGCCATTACTACAGGTCAGGGCTTCCAGACGGGATGTTTTGCTGTTCTTTGCAATACCGGTAATGTAGCTTTGTCCAAAATTGGATTCAGAAAAACCAGATTTCAGATAGCCTTTCTGAATGAGTGTTGCTGGCGTGATTGTTTTGGGTAAAGAGCCAGCAAGTAGTGTTGATGCATTGTCAGCAACATAATTACGAACAGCGGTGTTATAGGTATTTGCGTGTTCTGCTGCATTCTGCCAGCGAACATTATTCAGATGGTTTTCAATGTTTTCATAAACTTTAGGGATAACCATCACCATTATGCCGATGACAAGCAATACCTCAAGGAGAGATACACCTTTATCTGTCTTTTTCATTGCTCTTAATCCTCAGAATAATATTCCCGGTACGAGTGAGTAAACAAACATTCCGCCACAAAGAAATGGGCCAAATGGGATATATTTTTTTTGCTTGTTTAATCCGCCAAGAGTGAAGAAAATAATTCCGCCGATAACGGCAATAAATACAATTGAACAGGCAATCTCAGGCGTTGGAAACCATACGCCAGTTCCTGCAATAAGTAGCACATCTCCCATGCCGAAACTTTCCGTTCCGTTTTTATATGCAAAGTAAAGTCTTACAAACAGCATAATGAAGAACATCAGTATTGCACAAAACAAACCAGACAATATATTGTTATTCCATGTCTGATATAACAGGCCGGAGAAGATAAATGTCACGGTATAAAGGCGTGGTAGTAGAAATGAAAGAAGGTCTATACAGGCCATTAATGTAAGGAAAGCATAGAAAATAATCACTATTATAGATTGAATCAGTGATAGCTGATTTAATGAAAGATACATAACGGCAAGCGTTGTCAGTATCAGGCAGTAATATTTCTCTGCTCCGGTTTTATAAGATACATTAAGTTTATTTAAACGGAATTTAACTTCATTGTAGGCGTAGCATTGACAGACGATATTTGCACAGCAAAGTATCGCCGTATTAAATATGCTGTTTAGCATAAGTTACCTTACCATGTATTTATTGTAGACATAGCGTGCGTATTTGATTCTTTTCTGTTCGTTACTTTCTTTGAAACCAGCGTTATAACTGCCAAGACAACTCCAGTTTATGCCGCATTTCTGAAAATGTGTTGCCAGAATCCACGCGCCAATCTGTATATTGAGACAGGGGTTATCAATAAGCTCTGATTTGTCTTTTATTACGCCAAGTTTTTTCAGGTTAGGGATGTGTGATGAATTAATTTGCATTAGTCCGTAATCGGTGCTTAATATTTTTCCTTTTTCTTTGTTGTGATTTATCCGGTCTGGTATGAGTGAACTTTCTACAGTGGCAATGCTTTTCAGAAGCAGGGGGTCAATCAGGTAACGTTGCCCGGCTTGTGCAAAGCAGGCATCAAATCGGGTCGTTGTCGCTGAATATGCGGGTTGCAATGAAAAGATTAATGATGAAATACATGATAGCAGGATATAAAGAACAGAACGCACAATACTCCTCCTTAAACATGGAAAAAATAAGCGCCCGAAGGCGCTTATCCGATGCACATGAAAAATCAGGAATCAGTGCTGAAGGTCAGCGTATTGCTGTCAGATGCGCAAACAGTTGCTGCACTGACGGTTGATGTGGAAGTGTTGTTAATTTTGGAGATTGCGCTGGAAGAACGCAGGGCGTTTACCATTGCCATGCAGTTTTTCTGTGGGACATTGGCCTCTACAACATTGAAGGAGTATTTATCCGAAGAGGTGGTGATTGTAACGGAGCCGCCCCACGGGTTTTTCGCGCTTGCGCCAGTTGTATCTGCGATCATATCGGATGGCAGTAATCCTTGCGCGTAGAGAGTCTTAATATAGTTACTGTCAGTATAACGCCCCTGGAATTTCAGTGATTTCATGTTGGCAATCACTGTCAGGACATTGTTTTGTTCATTGGATGATTGAATGTTGGATTGCACCATTGAATAGAGTTTATATGCACTTGCTGCAAGTACAACAATAACACCAACAACCAATAACACTTCCATCAGTGTTGCCCCTTTGTCCTGTTCTTTTTTACGGCGTGCAGAAAGAGAAGAAAAAACAGAACGCATATTTAAAATATTAATAGAAGACATAATAAATAATTCCTTATTTATTGAGAGGTTAAAAATATTAGGGATTAGTGTTGCAGGACGTTACTGACATCGTAGAGAGCAGTAATCATCATTACAATAAATGCGAAAACAATTAATGCGGATACTACAGTTATCCAGACTCCTGTTGATTTGACTTTTCCTTTTGCTTCCTCCAGCCATTTATCAGCATAGTTAGACATCATCAATGCATTGTCAGCATTTTCAGAATGAAGTAATAGTTTGTTAATGCATAATTTCGATGGGAAGTCATAACCGCAACTTTTCATTGCCATTGCAAGTGACTGTCCAAGTAAAACATATCTCCTGATTTCTTTTATTCGTTCGAGTAGCCATCTGTTTTGAGTGCTGTTTTTCTCCAGCCTTTCCAGTGCTTTTGCAACGGGAATATCAATACTTAACATGGAGGCCATGTTGAAGAGAAAACTTACACCATTAAATGTTTTATAAAGTGACCAGGGGGGAATGTAATCAAGATACTGACGGCCCGGTCCTGTATAACGCGCAAGCGAATATCTGATCAGGAAAAACAGTACGATGCAGAAGCAGAGAATATACGTTCCGTTTTCTATCAGGAAACCAGACGTATCTGTCAGTAATCGCATAGAACTTGTCCAGCGTTCTTTCGGTGCGAGAGAAAGGAAAACAGGTGTTATGGTATCGTGGACCATATACATGTTTGCCAGACAACCAAAAACCAGTACGGATGGATAAATCATCGACATAACAAGAGTCATTGTCATTTCAGAACGACTTCTGACAATTTTTATGGCCTGAACTAACCCTCCCGCAAGGTCGCCGCGAATTTCACCGCTGGATATTAATTGATATTCATCCTCCTTTATATATTTTTTTAATGCTGAAGATATTGCCCCTGTGTTATTCAGTGCATTAAGGCACTCACAACAAATATAGTAGGTTGCAGAAGACCCCGTTTTCTTCCCGTAGTCTGTTTCTACATCAATCAGGTGTGTAAGAGCACGCTTTACATTTATGCCATCATTTAACATTCCAGATAAATCTTCATATAAACTTATTCTGGAATACCTGAATGTGTATTTTGCCAGCCAGTGGGATATTCCAGAGAAATCAATTTCTATCGAATTGAGTTTCTCAAATATATCTTTTATCGCCATGTCCCCTCCGGGAGCATTGAGTATTTATCTTCGTCAATGGGGGATATGAAGTGTGCGGACAGCGGATCTACCAGGCCATGATTAATCAGGTGTAATAAGTGCTGATTACGGGTAATTCCTCCAAGTTCATAGTGCCAGTATGATTTTGCTTCCAGACGCCCGGACTGACGATATAACTGAAAAAAACGTGCGTCGGGTGAGACGACTTCTGCTATAACCGTTCTTCCCGTTACGCCTTTATAACAGTGCTCGCAACCGTCAAGATTGCGCAACCGGACCTGTTCCGGCAGGCAATTTTCCTCAATGAGTTTCCGTTCTTCTGTACTCAGTTTGTCTGCCATTTCAGCATAAGTGCGCTTACAGTAAGGACAGAGTTTTTGTACCAGCCGTTGACTCAGCAGACCGATAAACAGAAGAGGATCTGCAATTAGCCTGGCCTGAATATTCTCCATTTCAAGGCGCTCAATAATATTCAGGGCATCGTTGGCGTGTAAGGTCGTCAACAGAAGGTGGCCTGTCATCGAGGCTTTGATTGCTGCTAATGCCGATGCGTGATCACGAATTTCGCCGTTCAGTATCGCGTCTGGATCAAGTCGCATAGCGGATTTAATCGCGTCAACCCAGCCATCGACGGAATCCCTGACTGCCGTCTGTATTGCGCCGGGAATGCGGCCCTCCGGCGGTGATTCAATGGTGAAAAGGCGTTTTCGGGGGAGACGCATATTATCGTTATGGTTGAAACCAAACGTGCTCAGATAGGCTTCTGAAGCCGTTCTCAGAGTCGTTGACTTACCGGACCCTGTAGGGCCGGATAAAACGATAATTCCTTCCGGGCGTTGTAACATTCGCCTGATTGTCTGGATTTGTTGTGGCATGTAGCCAAGTTCTTCGAAAGTGGGAATATCCTCGCTGTCATCTTTAATCAGGCGGAAGACCGCGTACACACCACCCACGCACGGATAATGCGAATAGCGCGCCCCAAAAAGATTTACAGGCTTCAGAAAATTCTCTGCGATACGTGCATCCTGCGGTATTGCCGGGTCAAACTGCGTACCTTTGATAACATCGCTCATTCCAGAATAGGCAGCACCCAGCAATTCCAGCCCTTCTTTACGCGGGATGACATCCAGAATTTCCAGTTCCCCATGCACGCGTGCTTCGACATAAGTGAACTCGGAACCGTCCCGACCAGGTGTGATGTGCAAATCGGAACTTCCCAGGTTGTTTGCTTTCGCCAGGTAGCTAATGACCTTTTCCTGGTTGCTGTTTAAGTGTTGTGATGAGTTTGAATCAGACCTTCTTTCGCTGTCCTGGTAGCGGGATGCGATAACATCAAGAGTGGTCCAGGTTATATTTACCTTTCCGGGATATTTAACATTCAGAGTGCCAATATAAGCCTGAACAGCACGCTGTCCTTTACTGTTATCAGCGATATAAACATGAACAGGTTTATCGTCTTTTTCAGATATACTGACGTATACAACATCAGAAATATCTTTTCCGGGATAGTCTGCGTCAGAAAAAAACATTATGTGTCGTCTCTTTCAATAGCATTAAAGAATAAGCTGGCGTTTCTCTGTACCATTCTGAATCCAGACGCCATTACCGGTGATTGATGCCACCTTATAAGAAGTACCAGGAATACTTTGCCCGACCTTGACATCAGTTACTGACCCGTCCAGGAGAGAAATTCGTGCTGACATTTGTTGTGGTGTACCAAATATTTCAATAACACGAAGTGATTTAGCGGCTACACGTTGCGCTGGTATATCGGTATCTGCAAGAGAAGAATACCCGGAAGAGGGCGTTATTGCTGGCGTTGCGGACGCTACACCATTCAAATCAAGATTACGCAATTCACTGACAGCCTTCTGACGCGCCAGCTTTGCTTCGTAAATTACCGTTTCAGCCTGAATCTGCTCTACCTGTAAAATGGTCGGAGATGAATATTTTATTTCGTCTTTTGGCGTGCTTTCAGATTCTGTTGCGTAAGCAAGTGAACTGAAAAAACAGACAGAAATAATCGTTGGTTTAATTATTTTTAACATAGAATGCACCTTTAATCTCATAATTCAGTCTTCCTTCTTCCAGTTTTATTCCTGCATACTGGAAGCGAACGGCAGGTTCATGAAAGTTAGCGAAAAGTATTGAAGGCGGGGTGCTGGTCTGAATCATTAATTCATACTCGTTCCACGGGAGAATTATTGGACGGCCTTCTTCATCTGTTTTTGTGTTTTCAATTTCCTGCCAGGTGAGTTTTAACCTCATTTTTTGTGCAAATGTTGTCAGGCGTTCCTGAATATCTGTTGCCTGTGGCAATGTGTCCGGGGTAATTGGATCAGGTGAAACGTCAAAACTGACAGGAAGAGAAAATCCCCCGGCGCTGCCTTCTGGCAATACAAAAGTTGCTGTGGTTGTCCCCTGGAATATTTCTCGTATCCGTGTGCTGAAGTCCTCGACCGTAACGCCGCTCAGTTCCTTATATGATGTGCGTAACAGCCCGTCATTACCGGATGTGCTGCATTCAGCAAGGTCAAAACGCCATCCGGCGATCGATAAGGGCAGTGCATCCCATTTATCAATGCATTTATCAATAAATGGTTTTATTTCAGGTAAATGTTGCCACGGTGGGGCAATCTGTATTGCCTGTTTGTCTGCCATTTCTTTTGCCAGACGAAGACGTGCAGCTTCTGCCGCAGCTTCTTTTTCCCGGTATTCCTGGTACATGGTGATCCCGTTCCAGAGCAGTATGGCCAGAATGGCACTTCCGCCATAAATCATGAACTGACGTTTTCGGGATACGCGAGTAAACGCCGCCTCTTTCGGCGGTTTTTTTACGTCAATCAACGCAGACAGAGTGAGTGAGGGGAGTGCCTGGCTGATATCCCATGATTCAGGCTGGTATAGTTTCCAGCCGGGTTCTGGTGTTTCATTAAACTCAAGAAATGTTGTCAGAGCACTCATGATCTGACTTTTGTTACCTACAACATCGTTGACGAGAACGTTATTGACGCAGGAAACAAAGCCATAAAGATCACCGAGTTCGCAGATAGCGTATCCGTCAGGTTCGATGAGGGGTTTTATCATCAGTGCCAGTGACCAGAATGAGGACGCCCCTGTGGGAAGATCCTCTTTTCTGATAAATGCCACCATCGTTTTGGTTTCATTTTCTGAATCCTGATACTGACAGGAGAGAAAATATTCAGCGTCGTTTTTTCTTGCGAATGTACGCGCATTCTTTTCTTCTGAAGGAAGCGTTTTCCAGTCCAGACCGGAGAAAAAAGCACGCTTGTTTTTAATAACCGAAGTGAAATTCCGGTTATTCTGATTATTCTCAGACAAAGTGACCTCCAAAAAAACTCCCTGTGTTTTTTCAGGGAGTTTTCAGTTAATTAGTTCACATTTTTTATTACTGCACTGGAGCCGGAATTAAGCACATTTGGTGTAATCAAAACGACCAGAATCGTGTCGCCGTCTTCGCTGTTAATGCCACCCCCAAGTCCAAAGAAGGAGGGGGTTATTACACCTTGCTTACTGGTTTTCCGGTTTGATTGCTGGAATCCACTCAGAATAACTGTCTGGCCTGATTTCAGGTCAATAGTCTGATTAATTGTTTTCAAATCCACATTCGGTGTTTGTGCTTTACTGCCGCCACTTTCGAACACCTCAAAAGTAGGTTTGTCAGACAGACTCATTGAGAACAGCAATTGCAGGTTTTGTGAATCCGGCTGAATGTATGGAAGCAGAGTCATATTGAATCCGGTCGTTATTGTTGCCGCATTCAGTGACGTGGATGATCCCACATTGGCGGTGGAATCGGTTGTGACACTTTCAATGAAGCCCTGCTGATTGGCAATTTGCATAGGGACGGGCGTCAGGTTTTTAGTCACAGCGGAGTTTTGCGTTACGACACTGACGCTGCCTTGTGACGACAGGGCTTTCAGAAATGCTTTTGAGCCAGCCAGCTTACCGTCCACGATGGATACACCACTGGTAATAACATTCTCGGCGGCATTACCAAATGAGCCACCAAGACTCAGACCAAGATGACCATCATTAAATACTGCGTTCCAGTCGATTCCCGCCTGTTCCTGGCTTGATTTCTTGATACTCAGGATTTCAACGTTCAGCACAACCTGACGACTCATTTCATTATTGCGCTTGTTCACAATCTCCTGAACGCTGTCCAGTACATCCGGGGTATCTGTTACTGTCAGTGAACCAGTGGACAGATACATGCGCCCTGTGCCTGGTGTCAGCATTGAGGAAACTTCGCTTTTCAGGTCGTTGTACAGCGATGATTTCATTTCCACGGTTGTAGTTTGCGTATTTGATGCGTCGCCAGTCATACCGCCAGAGCTGGTAGAGCCGGTACTGCCGCTGGAAGACATTGTGCCGCTCACAACTTTTGAGTTGTATGCCACATTGCTGTCCATATATGTGATCGGGAATGAGCGCGTTTCCAGATAGAAGAATGTGATACGACCTTTATCGTATTTCCAGGATATTCCCAGCCGTGAGGATACGGTTTGCAGTAGGGCAGGAAGCCCGGAGATATTCAGTTCCGGGAGTGATGTACCGCCAGAAGAGACTTTTACTGGCTGACTGCCTACTGACGCCAGAGGAACCATTCCATTTGCATCCGGTGCTGGAATTGATCCTGTCATTTGTTGTGTTGAGCCAGTGCCTGTACCGTTCAGATAGGTCCAGACATCGGCGCTGATTCGTACCGGGATATGACAGGTTTGTGTGATGCGTTGTGCGATCTGGCGTAGCTCAATATCTGAACGAGTATTAATCACAATCGGGCAGGGCGGCGCTGATTGCTTTACCTGCTTTTCAATAATTGGCGTTGTATTAATCCATTGTTTTGTTGAGTCCTGGATAACCGGGTTATTACGAATTTTTTTCGCGTATTCAGTCGCTTTTTGTGCGTCTTTTTCGGCTGCAAGTTCATTAGTATTGATTTTTTTGAAGTTTGCACAGCCTGATAATAAAAAAGAGGATATTAATAAACAAAGCAATTTTTTATTCATATTTAATTAACTCCGGTACTGGAATGATAACTGTGCATCCTATTTCATCGTGAGAGCGGGTTAACGATATAACTGTGTCGAAAAGGTTTTGTATCTGCACGCCGCCATCTGAAGGAGTCGTGACAACAATAAGACGTTTGCCACGTTTCCTGGCTGTTTTAATAAATCGAACAATAAACTGAACAACTTTTGATTCCCGTAAGTTGTTACCGATGGCTACACTATTAAGAACCAACGTTTTTTCAGGTGCAGATAAAAAGTCAAAATAATCAAAATTTTCATCGCAAAGATAATGGTGTTTTAGAAGGGGAAGGTGTTCATGGCGTTTATAAATATCACAGAAATCAAAATATTTAACGTCAGTGTCATTGATTAGCAATTCTGCTAAAAGTGTTGATTTTCCAGTTCCAGTTTCGCCTATAATCAGAATATGTTTTTCAGAGTTTATCAAAACATCAATGGCTTCTTTCATCATATTCATATTCATATTCATATTCATATTTATCATATTCGAGTGTTTTATTCTCATAATAGAATCCTGTCTTTGCTAATCAATAGTATCTTTATTGCGAGGATTATTCAGATACGTCTGTTTTATCGTGATTATCAAGGTCTGGATTAGTAATCACATTGCCTCCGGCAACCATGACTTTTTTTCCTCCAGCTTTAGGGAGATAAATTTCTGCAATTTTTGCTATTTCATCCATGACTTCTTGTGTAAACAGGTTTGGAATGTCAACATGATTGTTACGGGCGATATCCACGCGGCGTTTAAACTCTGGTTCTTCAAACCAGATTATTTTGTTGGCAATAAATGGTCGGCTAAATTCACTGGTTATAATTTCACGGATTGCAATATTTCTGGCTTTATTCTTCTTATCCCGGAGAGACACAATTTCTTCAGGAAGAAGAACCGGGCGCTCTATTACATCATCATTCCGTGTACGTGAACTTCCTCCCTTACCACCACCACTGGATATTGAGCGTTTGGATATTTTCATATCACGCACCCCAATCTCTTCTGAAATCTTTTTAGCCAGAGCATTTTTTGATTTCGGCGGATAGTAAAGCACAACAGCACTGTTTTCAGTGAATGTGGTCCATCCTTCCTGACCGTACATATCGCTTTTTTGCCCTTGTCCTTCATTCTGAAGAATGAACATAAAGCGAAGGTTATAACCTGCTGTAAAACCAACAGCGCGTTCAATGACCTCCGATTTACCCATTGATGTAAATTCATCCAGGAGAATAAGGCACTGGTATTTCAGGTCCGGGTTATGTTCCGGCAGTTCACGACAGTTTTCATTAACCAGTAATGAGAAGAAAAGGTTTACAATTTTCTCATGAGTGATTAAGGCATCCGGTGTCAGACCAAGATAAATTGACATGGGTTTTTTACGAATATCCCGGATATCAAAATCACTGAAATTCGTCGCTTCAGCAGTAACAGGGTTGCTGAATATATTCAGTGGTGATGAAAAGTTCGTTTTTATACTTCCCCTTGTGCGATCTGGTGCTGACATGAACTCAAAGAAGAATGATTTTGTTTTATCGCTAATCCAGCTACGATTTTCAACTTCCTGTCCCATCCATGCGGCGAGATCTTTTCCATTATCGGGAATCGACGTTTTCAGTATGGCGCTGATGGAAACAAGGACGTCCGGTGCATTATGACCTTTGGCTTTCTGGTCCAGGTGAAAACGCTCCTTGTCCAGCAGGTAAAGCCCTAACCCGACAAAAAGGTTGCGTGCAGAATCAGACCATATAGGATCATCACTTGCCGGGATAAGGATTGCGGCAATTTTTGCCAGGTCCGTTTCACGAAGTAAATCACTGCGACTGACGCAATCCAGAGGATTCCAGCGGTGAGAGCGTATCTGTCCTTTAATTGCCTGGTCGATAGTTTCCGCGTATCCGGCGGGCGCAAAGAGAAAGCATTTTTGCCCTAATTCTTTTTGCCTGAATCCGGCTGATAAAAACCAGTTTTCAAGTTTGATATCCAGTATGACCATACTTCCTGGATAGTTTACGCAGTTGGGGATAACAATCCCCACCCCTTTACCAGAACGTGTCGGGGCATAAAGAATTAAAAATTGTTGTCCGGCGAAATAAATAAATTGTTTTTTGTATCTCCCCTTGAACATTTTACCGATGAGTATTGGTGGATGTTTGTAAGGTGATTTTTTATCCGGGAAAAAACCTGATTTACTCAGGTCCATATCAGTTGCGAGCCGGGCGTCACCGTAGATGACTTTTTTAGGCATAATCCCAATCACGACAATTAACATGAATATGGCAAAGAATATTAATGGAACGCTGAATCCAAACCATGCCATAAAAACGAGTTTTTTATATTGTGGGTTGCCAGAAAATGTTGAGATTACAGAATGGAATGTATCCCACTTGAGCAAAGACATATCAACGGATGAATATCTTAATGCTGTGTATCCACCGAGATAGTTTGAGGCTATAAGCACACCCACAAGAAGCAGTAACAGGAAGAGAATTAACCCCCCCATCTTTTTTGCATTCATAGTTATCTCCACAATTTACATTGTGCTCAAAAAACGATTTTTTTCTTCGGGTTCGTAATACAGTTCAGTCATTCTGGTATGGTTGAAAAAAAGAATGACATCAATTGATGTTTTTATCGTGCGCATGATGAGGTCCATATCAACAGTCATACCAACATCAGATTGTTTTACAATACTTGCCAGTCGTGAAAATGAGGCATAAGTATTATTAGCATGAATGGTACTGATTGAGCCTTCATGCCCTGTATTAAGCGCCTCCAGATAACTCCATGCTTCGTTACCCCTAAGTTCAGCAAGAAAGATGTGATCTGGCTTTAATCGCATACACGCTTCAATGATTTCCTTCGCTTCAACGGTATTTTTTTTGTAGAAAAGCCGGATGTGGTTTGGATGCAGCGGCAGAGACATTTCCGGTACATCTTCAACGGTTATATAACGCCGTTCAGGTGGAAAGATATCTGCAATTGCCTTTGCTATAGTTGTCTTACCGGAGCCAGTCCCACCTACGATTAAAAAATTAAGACGGTCTTTAACTGCCTCTCTTAAAAATTGAGCTTTAGATTGACCATCCGGACGCCGGGATAATTCATACAGGTAGCGTTGCCGTTCAGTCAGTATTGCTTCGTGTTTTGTTGCAATCCTGACATTATCAAAACGCCCTGTTCGGACATAATCATCTATAGTGAAGCGTGTTAATGATGGCTTGCGGATAGATATCACAACGCTGTTATTTTCCGTTGCAGGTGGAATGATGATTTGTCCACGTTCCCCACCGGGAAGAACTACCGATGCAATGGGATTATCATGGCTAAGTGGTATTTTTATCTTTGATAAGTTCGTCAGAGTTTTGGCAAGTGTCATTAAATTATCAAAAGTCGCGTCTGGCGCATCTTTAGATTCCCAGCCATTTTTCCCTTCAAACCATATTGTTCCTGGTTTATTCACAGATATTTCTGTGACATGTTCAATATTAAGAACAGACTGGATACCCGTCATATCCAGATATCGTCTTGCGGTTTTACTGTTATCTCGTGGTTGTGATGTATTACTGGTATCTCTGATGTTATTCATATCAATTGCTTTACTGAATTACTGAAGTTCATATACGGATGAAAAGTCGATATTTCGCGGAACAATAACAGACAATACAGTCCCCTGATTAATATATGCCGTGGGGGGAATATTTATCGTATTGTCCAGCGTTGTTTTTGCCATTTCTTTTGTTGCGTCAGATGTGTTTTCGTAGGTGATATTGCTGTTTGAGCCTGTTTGTTGCGTGCTGTTTTTCAGAATATCTAAACCATCACCAAGTAATGACAGCAGCAATGCACCACCAAAACGCTGACCAAAATGATTATCGACCCATGCAGGAAGGCCGGAAGCGCCCAGACTGTCAGTGCCAAGTGCGCCAATGCGCACATTGACATTTTCATCTTTGAGCGTTGTCCAGTTAACGAACACACGGGCTACTCCCTGTGTCATGACTTTATTCTGCTCCCCGATCAGTAATGCTCCTTTACGGGCAAGAAGCACTTCGCCGTTATCCGACCAGACGTCTCGTGTTAACTGACACATTGTGATACCGGGATAAGAGGTTACGATTTTCGTTTTGAGCACACAGGATAATGCTGTTCCCGCAGATAACAGATATCGGCGGTTCAGCACAGGGGATACCTTTCCGTCTGCGTACTGACTGCCCTGTAAATCATCTCTTTCCTGGTTTTTTTCGCCTGGTTCCTGATTGTCGATCTTCACCATTGTGTCGCCCATCAGTTGTCGGACGGATTTTGGCAATGGTTGTGGTTGGTTTGGATCTCCACTGGCGGTTTGGGGACTGCCGTTGGCTATCACATTGACGGCTGTTTGATCGGCCTTTTGTGATGCTGGCGCTGACGGGGCATTGTCTGCCTGTTTCTGTTCTTGCTCCTCCTGTGCTTTCCTGCGGTTAGCGGCATCGAGTTTTTCCTTTTCTTCGATGTTTTTCATCATTGTTGTCAGTGAAACGCCATTGTTGGTATTTGTGACCAGTGTCTCGTCAGACTTCTCAAGAGGTGTTTGCTGAACGACCTGTTCTCGTGAAAGAACTTTAAACAAGATAATGATCACAAATAACGCGGCAATCAGTAATATGATGAGGATTAATATTTTTTTTGGTGCTGCTTTACCTTTTACTTCGATAATTCCGCGATTTCCCGCGTCATTATTTTTTTCATCATCTTTATTTTTCATAGCTGAACTTCTCTACTCGTGTTTTTCCGTTTGACGTACCTTTTTTATTCCACGGACGATTAACGATTGATGATGTTCTGATATCTGCAACCTGATCACCCAGGCGCAAACGGAGATTTTCAGCCACGTCATAGTAAACCATTGTGTTTTTATTTCTGTCTCCGTTAACCAGATGTTCATTTCCATCGGCATCAACACGATAAAGGACCGGAATTTCCTGTTTATTAGTCCATCTCATGCATGTAAATTCGCCATTATCCCATACCTGATAAGGGAAAATTGATTTGTCCCCCTTGAGTTGATAATGACCATTAATTATTCCACCATCAGTGCATGGAAATGGCTTTTCAGTCATTACGGTAGCTGACGGTGGTTTCGGATAATTAAAACGCAGAATATACGTGGGTTGTTTCGTGGTTGATATCAGGTAAAGAGGATATATATGCTTATTGGTCTGAACGGTAACGTTTGTATCCGGTTCTTCTGCTTTTGGACGCAGAAACAGAGTGTTGTCCCTGACCGATACTGACCAGGCTTCTGGATCACCAATTCCGACATCTTTTATTGTTTCGTCTTGTCCGAACTTAATTGTTGTTTGTGCGCCTGCTTTTACGCGCACATTAAATACATCCTGCTCGTTATAATCTACGGTCTGAATACGCCCGTCTCGTTCCGATGGTGTGCCGTACATAGCTGCTAAAGCACCAGGTACTTGTAGCATCGACAAAAGAACTATCGCAGTAGTGCGTTTAAGCATCATTTTATAACCTCTGGATCAGGTTGGTAGCTCAGAACTTGCAGTCCCAGCGGATTCACAAGGCGCTCTTTTTCTGTTTTGATATCTTTATCCCAGTCAAAAGAAATCGTCGAAATCCACTCTGTTGCTCTGTATCCGGGGGCGGGTTTACCAGATAAATCCAGTACCATTTTTTTGAAACGCACCTGCGCCATATCCTTCCTTAGAAGGATTACTGAATTAATCTGGACCTTAATTTTGTAGTTGTTTTTCAGAATGTTCAGTGGTGAGGAATCAGCACGAATCATGGTGTCATAAGCTGAAAACACCTTTTGGGAAGACATTGTTTTTACTGTATCGGCCTGTTCCTGGATAGTCTGCCAGTCGTATGCTTCATAGTTAATGAGATATTTTGACAGGAAATATTTAGTCTCAACATCCTGATAACTTTCCTTAGCATCAGATAATTGAGGGGCAATATCAGTATATCCAGTGCTGTTATCCACTCGCACAAGGAACGGTACAGCGGTTTTTAATGGAGCAAGGGAGGCTATTGCGAAACCAAGAATGCAAATTACAACAAATTCACCAATGGCGAGAATGGTAAATGTTTTTGCCTTTTTCTTTATGATTTCAATTTCACTACGCTCAAAGTCACGTATGGCCTTGAAATAATCCTTTTTTTCGTTCTTTTCTTCAAATTTCTTTTTGGAGGCTTCTTTTTTTGAATGTTCATAATAGTTTGTGTTTTCCATTAATAATCACCTGCTTTGATCTGTTCAATTGCTGTGTTTAACTCAAACCATCCCCCTTTTTTCTGCACAGGATTGGGGGTGTGAGAACATGCACTAATTAATAGCGCGGAGGCAATTATGAGGGGGATTTTCATATTGTTTTCCGTTCTGTATAAAACAGAGAAATTAACCGTTCAGGCGTTTCAGGAGTTCTGGTATCCAGATATCAGGAGCTACATCTCCTTTTTCAGCAATAATCTCATCAAAAATAGACAGGCCAGTTTCAGAGCCAGAAAGCACGGGGATAAACTCATCGAATCCAAACAAATCCATTTTGGCGAAAACAGAAGAGCCAGATTGCTTGATGAGCATGGTCCTTGACTCCTTCGTCAGTTCTTTGAGTTTTTCAAACTCTTTTTCAGTAAGCCCAATTTCTTTATATCCTTCCCATTTTGCATCGGGATTGGGCAGGAGAATTTTCGTTGCGGTTTGTTGTACCAGCGCCGCAAAAATTGCGCAGTTTATGGCGTCTTCCGGGGACTGTGATGTTAACCACATCATTTCACCTTTCATGCGCCCCGCTTTCAGTGCGCTTTTAATCATGCTTTGTGTCATGGGAAAGTTTGCTGGCATCCAGAACTCTTCAATAATGGAGAGCATCAGATTGCCGCCGCGTTGCATGATTTCTTTGTAGAAAAACAGGACCGCGAGTAAAGGTTCACAGGCAGGATGAATACCGTTTTTTGTGTCCAGGACAACGGTTGTATCAAATCCCACTTTTTTATGATGCAGTGGATTGAAGGTATTCCGGGGGGAATCCACCGCCCAGGCATATTCGCCGTTGTCGCACCATTTGGCGAGACGCGTCTGAAGTTCAGGACTGACTATATCGAGAAGAAGGGCTGTCCGGCGTTCTTCGACAGGCATTCGCATAATGCTTTCCACGGCAGCGTTTAATTCAATGCCATGTTCATCAGAACAGGGGTTGCCCTGGTTGTCTCGTGCCAGAACCTGTGTCCAGCGTTTCAGAAATGCGAGAAGTCTGTGCCAGACGGGAGATTCCGGGCCTTCTGCCAGTTGCCACGGATTGCAGCCGGTATAGATACCTTCCTGCAATGTGAAATAGCTCCCTCCGTATGCGCGGACAAAAAGTTCGGTAGAACGGTTGTAATCCACAACAAACATTAAAGGGTCAAAGCGTTGAAGAAAGCCGCTGGCTGTTGCTTCAAATGTTGTTTTCCCTGTGCCTGTTGCGCCAAGAAACATTGCGTGTCCGGCAATTTTTTGCCCGGTAACATTTTTTTCCGGTGGGCTGTAATGTGTATTGAACCAGTAAACGCTGCCTGAATCCGTTTTTAAAGGCATTATGGCGGAGCCATCACCAATAGGATTGCCTGATTTTTTCCCGGAGGAATAATTGTGAAAAGACATCAGACAGGCCAGGTTGGTAATTGTTCTTCGGGTATCCAGTGGACGCCTCTTATTCAGCGGCATATGACTGAAAAAAACAAAAGGTGATGCGAGTGAGGCGCGACTGAAGCGGAATCCCTTACCCGAAGTAATAAACTCAGCAGAGAGCTTTATACCATTGCTTCTGGCCTGATCTGGTGTATCACCGAATACCGTTAATGCGCAGTGTAGTGAACCAAACAACGTAATTCCGGCTGCGACCGCTTCTTTACCAGCTTCGAGTTCTTCCTGCTGTATTTTGGCTGCATCATTAGCTGATTGTAGTTTGTTTAACTGTGAATCGATATTCTTCAGGGTTTTTGTTGGTGACTCAAAAATAAAGGATTGAGTAAGAATAAATTCATAGGGTTGTTTGAGAAGGAAATCCCATTGTCCGGGGGTTGTTTCTATCGGGAAATCCTTCAAAATATAATTCGTTGCGAATTTTTTACTATCCGATTCGTTGTTACGCAGTTCAAGAACATCAGCACCAAAATACCATTCAGAATCACAAATACTGGATGATAAGGGGGTGGATGATAAAGGAATCATGTTATGTTCTGCATTTAGCAATAGTGATAGATAGTCTGCCACCTCACTAATATAAGTATTGTATACGGATAATAAAGAAGCGTTAAATGGTAGAAGTGCTTTTTGCGCCTGTTGTGTCATTTCATTCATGCGCTCGATTCCGGTATCAACATCATCGTAGGGAATCCCAAATGTCAGATACCATGTTGATTTATAGAATGCGCTGGATGTGAACAGAGCAAGGTATTTTTTTGAAAACCTGCTTAAAAAATCATTGTCAAAATTCCATTCTCCATTGATGGTTGCTCTTTTCTTGATAAGATGAGTCCATAAATACAGGTCGCCTTCCTTTCCTAACCCAATCAGGAAGTTTTTAACGCTGTTAAATAAATTTGTAAGAACATTATCGTTCTCCGTCTCAAATGGAATGCCTGCTACAACAAGTGAAGCCAGCATTTTTCTGTCAGAAGTAAAGATAATACTGTCTGTTACATGGAAGCGATAATCGGGATATTCACGAGCAACATCCATATTGTCACTGTTTTTTAATTTGATTTTGTGCATATATGTTACCTGACAGATGAATAACCAATAATTAAATCTCTGTGGCGGATTTTTAACAAAAGCCCCATAAGGTTTAAACGGATGACTCTTAGTGCGTTTGAGTCATTTTCACAGGCCAGTTTTACTATAAAAAGAAACAGCGCACATATGACAGGAATAATTATCCCTTTTATACCCCAAAGGAAAATAGCGGGGAAGCCTGTCAGAACAGCGAAAAAAGCCGTGAACAATAACAACATTAAGGGTATACCAGCAATTAACGCAGGACGGTTAAACCCGTTATAAGTCATGAATGACTCACTGACATGTTCATTTACTTTGTCCATAACTCACCATGAAAAAAGCCGCAATTCACACATAAGGTAAATATGCGGCACAGTTGTATATATGGATTACTTATTTGAAGAGTTCCAGCGCCCAGTTCCCGGCGTAAAGTGCACCGCCAGCGAGGGAACAATAGACAAGCGCCATTCCTACATCAGACCAGCTTTTCTTCTCCATGAATGCCATGATGACGTTATAGATCAGGTAACACAAAGCACAAACACCACCAAAGCTAAATGCCCATGTCTTAAGTTCTGTCATGGTATTTGTTGCGGTGTCGAGTCCACCTGCATAGGCAACGGAGGCGGTTAGTGACAGGAAAAACGCCAGGATAAAATACTGCAAAGTTGATTTGACTTTCATTAATTAAAATCTCCAAAAACATCCCAGTCCGGTGATGTTTTCTCTTCATCATTCGGGGGTTTTATTTCTGATTCATCAGTTTTTTCACGGATTGATGGCACGATATAGTTATTCGCGTTACGAATAACCCGTTCCAGATAAGAAGTGTTGTTAAATTTCTTTTCTTTAATAAATCCTGTTGATTCGTTACCCGAATAATACAAAGATAAAGCGCGGAGAATTTTTTGATTACTACTACCCTCCTTTTGTCTCAGATAATTGTCCGTCAGGACTGCCGCTCCTGCTTTTATATTTGTACACGGGTCAAAAACTGTTTTATTAGTCAGATTGAGTGATGGGAAATTTTTGCTGTATATCTGTGTCAGACCTGCACTAAATCTTTTATTTTCTGCTGTTAATTTTTCTGCATATTCAATAGCTCCTTTTTCATCTTTAAAATACTTACTTGTTCCGTCAGAAACATTCGCTATTACATATGGATTAGCCCCTGATTCAGTCATAATCAGGGCATTCATCGTATCGGGTGATACATCAGGGGCACACTGACTTATCAGTGCTGCCAGAACAGCCGCTGATAACTGCATAAGGGATTTATCTCCTGATATTAAATTCGTGAAGAACGTTTTTTCCTGCGGGGGCGACGAACGATACTGTCCAGCTCATTAGCGCCGGAAAGAAGTTCTTTCAGCGAGACAATTTTTTCCCTGACAGGTATAAGGGATTTTGCCCCCGGACATTGTTCAAAGTCTGATTTGTACAGGTCGTATAAAACAAGCGTATGTTTTACATCCTGTGACAGATAATTATGTTTATTCTGGATGTTACCTTGTTCGATATTTTTGATAACATCTGCTGAACGATGAATTAAATCTGTATAGATTCGGGTGTTTTTAAGGGGATTTATTTTTATATATTCAGAAGCAAGCTGAATAAGATTGCATCGTAAGATAAGGACTTCACGAGTGGCAATATCAAGCTCGCTGAGATCATCATTATCCTGCATGGGTATCTCCGGTAAAGATTTTCTGGACTGAAGTCCGCGCCGTAGCGATATATCCGGCCCACATAATGATGTGTCGGATATGCAGACTCCAGTTCAGAATCCCCTTGTCAGGGGATATTTAGTTAATGAGTACAATAGCCTCTTCTGGAATGGAGGAAGGAACGGGAACGCCCATATCATTCCCCTGATTATCGACAATCTTCTTATTTTTTACTCTTCCTAACATTGCTGAATGTTTTGTTTGCTGCTGCAAAGCAGACATCAGACCTTCTGTATCGGCAGACCAGATATAAACTCGCTTACCGTAAATAATATGATGAATATCATAAACGGGTGTTATACCGATTTGCTCAGATGTTAACGTTCCTCCTGAATTACGTTTGTCTGGATATTTATAAAGATAGTCATTGATAACATTTGCGAAATGGACAAATTCAATTGCTTCCTGTTTTGCAGGAAGCAACTTCATTCGCTGTATGGTTTGGTTATTGTTATCTGTCAGATAGTAACTGCCTATTGACAGGAATATCAGGAGCAAGCTGAAGAACCAACCCATAGCGATTACTCCTGTTGTGTGTCTTTAATTGTGATTACGCATTGTGAAATATTCCTGAATGCGTATAACGGTTTATCTGCTGAAAGATAAAGCATCAGCGTATCTTTTAATGCAGATTCAAAGTTTCCCCGGAAAATAAGAGGAGCATCTATTCTGTAGTTCACAGGAGTATCCCATACAAGATTCCAGGTTGAATTACCACACGGTGAACTTGACGCCCATTCGGTGAGGCCATCCCGCAGCGTCGAACCTTTTTTAAGATTCCAGGTGAGTTTTTCTGTCTGTTTTTGCGGAACGGTGACAGGCGTTAGCGGTTTTTGAGGTAACTTGTCAGAGGAAACTGGTGTCCTGACAGGAGATGCAGGGGTTTTGTCAGGAGATACAGGTGCTTTTTGTGATGTTTTCCCTTTCTGTTGTTGAAAATCAATCAGAAGATGTCTTTTTTCGGTGTTCTTAATTACCGTCAGACCGTAGTTGCGAAGCATTTTATCCAGGACATAAGGCCATTGATCGTTGCCCTGCCAGGATATCGTGCGACGGAAAGTTTTCGTTACCTCCGGGGATATTTCAGCCGTCCAGTCCGATGGGACAATTTCTTTTAATGCTTTTTGCAGCGAGCCTTTACCATCATGGCGAACAAAGTAAATTTGCTTTTCAGGTGTTGATGGGCCTGTAAGCGTGGTGGCCGGAACAGCGTTGTTAGCGTTTTTTTTCAGAAAACCGCTTTTTGACATTAGCGGTGGTTGTTCAGGACGCGGTTTTGTCTGCTGATCAGTCTTTTGTGCCGTTTGTGCCTGATGGTTGTTGCTACTGATTGGCTTTGCGGGAGAGAGTGCTGGTTGTTCAGAATGCTGTTTAGTTGCTCCTTTGGCTGCTGTTTTTATCTCACCAGATGAAGTTATGCTGTTTGTTGTCGGCTTCAGAAAACTATTATTGCTGCCCTGGTTTTTTGGCGCTGTCGCTGATTGCTCAGAACGTGGTTTTACGGTTTCTGCAATACGAGGCGCGGGAGTTACGTTTATGGCTTCCTTTTTGAGATAATTATCATTGCTGTATTGAACAGTGCTGTTTGGCTGTGACACGGGCTGCTGAAGGGCCACCCCGTTATGGTATGTGTCTGCTCTTACCTGCTCAATTGTGGTATTCAGTTCATACCAACCTCCTTTTGCCTGTGCGGGTTCAGGGGCATGGGAACACGCATTCAGCAATAATGTGGTGGGAATAATAATGAGCTTTTTCATGGTTAACCCCAGGAACGTCCGAACTTACTCATGATTTTTGAGATCGCCGCAGGGTCTGCGGTCGTGCATTGATTAAGAAACGCTTTTCGGGCATCAAAGGTTTTGCTCCAGCGAATAGATCCCTTTTTCTTTTTGATGATATTAAAGAAGTCCTTTTCGGCGCTTTTGCATTCGCTCCCGCCGCCGCTACCGATAGCTTTACCGTACAGACAAAGAACGGAAGCACACGGATCAGACGCATATACGGGGGCAGTGAGTGCGATAGCACTTGCGGCAACCATCGATAAAATGATGGATTTCATGGTGTTCCTCCATATTTCAGGCAAAAGAAAACCCGCACGAGGCGGGTTACTTGACAGGTTTAAAATATCAGCGGGTATCTGCCATGTGATAGACGTCGCTGCCTTCTCTTTTTCCCACAGAAATCACAAGCAAAACGACCTTCTCATCAATTACCTGATAAACGAGCCTGTAACCGGATGCGCGAAGCTTAATTTTGTAACGGTCAGTATGACCATGTAGCTTGGCTGCGGGGACGCGTGGATTTATCAGGCGTTCTGACAGTTTCTTTTTGAATTGCTCACGAACAGTGTGACCGAGTTTTTGCCATTCCTTCAGTGCTCTGTGGTCAAATGCCAGTTCATAAATCATCCAGGTTAACCCTTACAATGTCTGGATTTTTCATTCTTTCATCAGCAATTGCGTTGAGTTCAGCGTCTTCAGCGAGTTCCCGGTAGTAGGCATAAAGTTCTGGTGGGACACAGTAGAACGCTGGTTCATTTCGGTTCAGGATTGCGACGGCATCCCCTTCTCCTTCGGCTACAGTTCCCATAGGATTTTTTTTCAGGTCAGTAATACTGGCTGCTGTCGTAGTCAGAATTTGGTAGCTCATAAGTCCTCCATTTGTATCATGGAGATTGTAGCACCTTAAAAAGACTTTTAAAGGTGCTTTTGATTGTGGGGAACGACAAAAATGATCCGGGGTTGGGAATCAACGCACGTGTGGCGTAATGATGACAATCCGGTTCTCACTTTGGCTATCAGCATTAACTCCAGTAAAACCACTCACGATAAGGGGCTGGTCCGGTTTGAGTAAAATATTCTGCTTGACAGTCAGTTTCCGTATCGGGTCAGTGGTTGTGAGTAGCAGGTGGAAATTTCCACGCTCAATCTCCGGTGCTATCCGCATTGTGAAGCCAGCAGTCCGATCTTTTTCAGCCAGTGTAGTGTCACCGGATATTGCCTGACGGGGGATCAGTTCCGTGGCAGGTTCCCAGCTTCCGGCAGATGTTGTTTGTGATTCAACGCACGGGGTACTCTGCATTATTTTGCGGATTGTTTCGGAGGATATCGCCTGACCGTCATCAAAGAATGCACTACGGAAAATCACGTTCTGATTTTTGGCAAACAAGCCTGGATGCCGCTCGCTCAGACTTTCGTATATATTCATACCATTCAAACAAACAGGAGAACTGTCCGGCAGGTCTGTTCGCACGGTATCAATTTTCAACGTTATCTGGTGGTCATTTGAGGTTAACGGTGACTCAGGTACTGGTTGTGTTTTGCTATCCGCGCAGCCTGACAGTAATGCGGTGGCGGCAACCATCGATAAAATGATGGATTTCATGGTGTTCCTCCATATTTCAGGCAAAAGAAAACCCGCACGAGGCGGGTATGAATGAGCCATTAAAGGCGGAATTGCACTCTTTGTTTAGTTTTGATATCTATCCTGTTGCATCATCAGAACAGAATACTGGAGTTAAGTTGGTTATGGCGCGGTTTTATTTAAATGTACCTTTTGAAGAAAAGGAGTTAGCCAAACAAAAAGGGGCGCAATGGGATCAAGAACAACGGAAATGGTTTGTTCCACAGGGGAAAAATCCAATTTATTTTATTCAGTGGATAAAAGAATTAAACGAACATGATTACAATATTTTTTCTCAACGTTTTTATATTGCGGAAAGTTACCAGTCATGTTGGCGCTGTAAGAAAATAACTCCTGTTTTTGGTGTTTTTTTGCCTCGTTGGTTTAGTTATAGAGATGTTGTTTGTGGGGTTAAACCTGCTGAATGGGAAGGTCGCATATTAGATAAATGGTATGAAACATCTTCTCCACGAGGGATGGTATATTTTGATTCAAAGAAAAATATAATTTATCAGTGGTTAACCAATCCAAAAGCATGGGCTATTTTATCCAACGTAAGAAGGATATCATCTTCTGCGTTATCAATAATCAATAAGCATTCTAAGTTATATTATCCAGCCTATAGTAAAACAGCGAAAATGACCTACTACGCGAATCATTGTTGTCATTGTAAATCAATGCAGGGAGACTTTATGATGTTTGATGAACCAGGTGGTGTGTTTTATCCAGTCACATCTGAACAAGCGAAGAAAATAAAACTTCATGAAGTAATAAATGAAACAATTTTTGCTAATGCAAACCACAGGCAAGCCATAGAGTAATGCTTGGTAGTGGAGGTTTTATAAATCTTTACGCAAGATAAAACCCGCGCGAGGCGGGTTTTATTATTGTTTCGCTTACTCATATTAATCTAATCGTGACTTGTTCATCACCTAAATGTCTCTCTCAATTTCTTCTTATACTCTTCTTTTTTTATCTGCTCTAATGCCGTGCGAGCATTATCACAATTCTGGTCTGTAATTTCGCCTTTTCTGCACTGCTCAATGGTTTTAGCAGCTTCGGCTGGGTTGCTTGTATAGTATTCAACAGCGTAAACTTTTTCTTTACATCCTGCTAACATGAATACTGCAAATAGCATGGATGATGCAATAATTACATTTCGTGACTTGTTCATCACCTAAATGTCTCTCTCAATTGGCGAACATATACTTCTTTTTGAGCTTTGCGTTCAGCTTCAAGCTGTCTCTCTGCAATATCATATTGCTTCAATTGTAGCTCCATTGTCAATTTTTCTGTATTCAATTGGTTAGATAAGTCAGCTTTTTTTTGCGGAGTATCTGCTGACGCGAACTCCTTCTGTAACGTGGTTAATCTCTTAGCCCTTTCCTGTATTTCATTGTTGAAGTTTTCATAGAATTTGATCTTAGCCAGGATTGCATCATATTTTTGTTGCGTCTCTGCAACGTTGCTTGATAAGTCATATTGCGATCGCAAAGAGCTAAGATCTGATTTTGCACTATTTGTTATTGTTGATAGAGAATCCTTCATATAGCTGGATGCGCTATCAAAGCCATTGCTGAAATCAGTGAAGCCTTCGAGTCTTTTCTTCTGTTCTTCAGCGTATCTTATGGTCTGTTCATATTGTTGCTTGAGTTGCTCAAGCTGTCTTGCCGCTTCAGCGGCACGATTTAGCCCTTCTTCAACTGTTTTAGCTATCGCTGTAACGTCTACTACTGGAATACCAGATGCAAAGATTGATGGTGATGTTACGGTAATTATTCCAGCAAAGAGAACGGACAGGACTTTATTTTTCATGTTAATCACCCCGGTTTAATTATATTTCCAGACCTCGGGGTTCGTCCCTGCAATGATTTGGCAGCGCCCCGTCCAGCCTGGTAAAATGGCATTGACATTTGTTTGGAGAAACCTCCTGCGAAAGCGCGAAGCCCTGATGCTCGCATAAAACCGGATGCAACCTTACCTCCAAAGCCATTAGCGGCTGATGTAAGGCCATTTATACCTACCCCACCTGTTAATGTAGAACAGAGCGTGCCAACCTGTTCTACAAGGAATACAGAGATAACAATGACCATAAAGAAATAAATCACTGTTGTGAGGGTTATATTTCCGCTTGACGGGACGGTTTGCTCCACAAAACTGATAACGAAACTGAAGGATATACTGTAAAAAAGATTAAGTAGAATGTAGTTAAGACAACTCCCCACCCATGCTGTAAACATATTTCTTGTTGAAGCAAACAGGGAAAAGCAAATAAACAAAATTCCGGCGGATAAGAGAATGCCAACCATGAATGTGGATAGGCATAAGAAAATAGCTGTGTAATAGATAAGTAGTAATCCGCCCCCATAACCGATGAGCCAAACGATATGCACATTTATTAGATCTCCGAGGTCACTCCAATCTAGAGCATCTAATGCTTTAATCTTATAATCGTTTAAACTCGTTGAAAGCATATTCCACAAATTATCAACACTGGTTGCAGAGCTTGAATTACCCGTTACAGCAGCAGAAAGGTCGGAGCCTGCGTGCATGACAAACGGAATCACAAACTCACTGTAATAAGGTGCTGAATAAGTGAATGCACCAACCAGGGCGAATGCACCGATATTCTTAGTCACCTCGCTCATCAGGACGTCACGTTGGGCATACGTGATTTCATATATTAGATAAATAACGTATAAGGCTATAGCCGCAAAGAATGTAGGAGATATTGCCTGTGCGACCTTAGCGGCGTTCGTCGCCGCTGTGGATTCAACAATGGTTGTTACTTTCTCAAGAAATTCTGAAACAATGTTCATAGCTTAGTCCTTCTGGCAGATGAAGAGTTCACCAGAGGCACTAATATTTGGCTTCAGCTTAATTACCAGGTCTGTGCCGAAAAAAATTGCTTCTCCAATTTTTTGGAAATTTAATGTGGATTCTTCTTTATTGTAACTATAAACGTTGTGGGTCGCTGTCGATGTTTTGGTTTTACGTGGTGTAAACGCTTTTTTAATGATGTAATCGAGAGCGTGGCTGTTATGAGATGGTAAAGTGATTAAATCGTCGTTTTTTTTCATAATGTAGTTCTGATTTTTGAGGTTGAAGATAATTGTCGGTCAATGCTTTTGATTTTTTTTCGTATCTCAATTGGATTCGCCTCAGATAAGTTAGCAATGGATAAAGCCAGTTCGATTTGTAAAATTTTCCTTGTTTCGATGAGATTGATTTTTACTTGTGGTATATCACGGAAGACACCACAGCCGGTAATTGCTTTTGAAACGACACGTTCTGTATTCGCTATGCTTTTCTTTACTGTTCGAGGGGGGACATAACGTGCACGGCGAAGTTTTCTCAGATTAGCTTTTGCTTGAGTCATGTAATGTGTCCAGAGTTATAATTTTATTTCTTTGCATGATTCCCCTTTACGGCACACAGATACACGGTTATTTATCACATTCACCACTGTCACAGTTCCGTCGTCGCATTGAGTGACGATGAAAGGGTATTCGTTGATTTTTGTACAGTGAGATTGTTCTCCTGCTATTGCAACCACAACCGGGCTAATAAATAACGCCAGAACAGATGCCAGCTTAATAAAAGCACTCATTTTTGTATGCCCTATATAAATAAATACTTTCTTCAAATCCTGAATATACTATCTGAATACAGAAGCGGTCCTGTTCTCCTGAAGTAAGGCTTCATTATTTTATGGGCCATTGGATGGGTAGTATGTACGGGTATTGATAGAATGAATTTTGAATGTCCTGTTTTTATTGCATTCAGAAGTTCGTCAATTTCTTTTTGAGATGTCGTTGCTTTCATCAACAAGTGCCTCAATAAGTTTATTAATACACGCCCGTTCTGCCGGAACCTCCCGGCCTGTAATCGGGTCAAAAACGACATGATCGCTTTCAACGTAACGTAAACGGTGATAGACATCTATACGGAGTTTTGTTTTAGCAGCTTCACGTTCAACTCGAGTTAACTCTTCTTTTTCTCGTTTGCTGCTGAAAAACAGAAACCAGATCATTGCTATGATAATTACTAAAAAAGGGATAACGGTGCCTAAAATAATTACTAAAAAAGGGACAACGGTGCCTAAAATGCTTACCATTTCTGCCATGCCATCCAGAATAGATTGAATCTTTCCTGTGGCTGTGGTGGTTGTTACTGGTTCAGCAAAATCAGAAACAGTGTCTGATAAAAATGTAAATAAACGGGCAGATAAACCAAATAGCACGCCAAAAACTAGAACTTTTATTATCGAACCGTATACATTTAATAGACTGTATTCTTCCTCTTTAGGCGATTCAAAGTTTTTTGCCCTTTGTGTCTGCTCTTGTTTAGAACCAGAAACACCTCCGATGAATAAGCCAATACCACCAGTACCTAAACCAATACCCCCACCAGTACCATTCCATTTACCTGTTGTTGTTTCTTTAGCTACAATAGCTGCAAGAGAAATTCCTTCATTTGCGTTTCGCATAGAATTTTCCTCATACTATTCCTGTGACACTTAACACCATCAATAAAATATGTAATGCAGTAATTTTTTTTAGCGTTTTTTTATAGACTTCAATAAAATCCTTATCCAGAAGTCTGATAATAAAAATACATAGTGCATTCATCGCTGCTCCTGTAAGCGGAATTAACAACGAAAGATGCATGATTTATTCCTTGTTTTCACTCTTGTCTTCGATGGTCATTACTTTAAGAGTTATTCCTTGCATGTCTGTAACAATAACTCTTGTTCCCGCAGGGTAGTTTTCATTGTGTAACGTCTCAACAGGCCAGAATGTATCACCAATAAGTAATTGCCCCTTGCCGTTCACAATATCGTCACTGAGAGTAAATTCTTTTCCTTTAAAACGTGAATGCCCTGTGTTCACCAGGAGAGTATCTAATTTCTTATGTTTTTTGTCATAGAGGAACTTAATTATCGACGCCAGTATGCTGATTGATGCAAAGAAGCATATATTGGCTTCTTGTGATATCGGAAGAAAAAAGCCCACAATGGCGCTTGATAATGCACCAAGACTGATTAAGAGTAACCATCCTGTTCCGGTTATAATTTCAAGTGCAATACACAGAAGAAACAGTGTAAACCATAACATGGCTAATCTCCATGATAAAAGGGAGAAATTGAATAAAGAAGATGAAGGTAGTTATAAACGCCAAATGCATAAATTAGCAGAAAGAAGCCAAAGAATATGCCATTAAAACTCCATATTAACAGGGGCAGCAGAGCCATCGCTATCATCACCCCTCCGAAGTTCAGCCATAATGGAAATATTGATGCGATGTGGTTTAGTATTTTTTCTCCTTTGTTCTCATCTTTTGTAATAAAAAGAAGAAAGCTCCATGCCCCCATAGGGATTATATGTAAAATAAGAAGGATGAACCCATAAGTTTTCATTTAAATGATTTGCTCATTCGTGAATATGAATATGAGGACGGTGATTAAAACAATATTGTCACCGTCCGCTATCTGTTTTAAGACAAATATTGTTGAGCCTGTTTCATATCAACTTTGTGAGTATCCTGTGATACGTTTTTTAGCAGTTCACTGATACCTGCAACCGAACTCACCAGGCTACTTGAATCAAGCGGCATCATAACCAGTTTACTGTTTGATGCTGTGCCAATTGCCTGGAGTGCTTCAGTGTATTTTTGCGCAATAAAGTAATTAACAGACTGAACGTCACCTTCAGCGATTGCATCCGACACCAGTTTTGTTGCGCGAGCTTCCGCTTCTGCCTGACGTTCGCGTGCTTCAGACTGAAGAAACGCTGATTGCCGTTCCCCTTCCGCTTTCAGTATTTGAGACTGTTTTTCTCCTTCCGCCTTCAGAATTTGTGACTGGCGTATACCTTCCGCTTCCAGAATCTGGGCACGCTTAGTACGTTCCGCTTTCATTTGCGCGTTCATCGCCTTAGTCAGTTCTTCTGGTGGTTTAACATCCCGAATTTCAATACGTGTGACCTTAATTCCCCACGGATCAGTTGCATAATCGACAACCGTTAACAGCTTAGAATTGATGCTGTCACGTTGAGAAAGCATATCGTCGAGATTCATTCCACCAACGACAGTTCGGATATTGGTCATGACAAGATTTGAAATAGCTGATGCAAGATTATCGACTTCATAAGCTGCCTTCGCTGCATCAATGACCTGGATAAAACAAACAGCATCGATAGTTACGTTTGCATTGTCTTTCGAAATAACTTCCTGTTTAGGAATATCCAGGACGGTTTCCATCATGTTGATCCGTTGACCTATGCGGTCCATAAACGGGATCAGGAAGTGAAGACCGGGGGAAAGCGTATGGGTATATTTACCAAAGCGTTCCACTGTCCAGGCATTACCTTGCGGCACGATTTTGACGGCAGATTTAACGAATATCAGCGCGACTAAAATTAATAAGAGTAACGGAATAGATGTAACTATTGGCGTAATTATAGCGTCAATCATGATTAATCCTCTCCCCATTCATCACAATATGCTTCAACCGGAGTTTTTCCTGCTTCATAATCACCACGCCATGCTTCCGCATCGGCGGCATTTCCACCGCGTAACTCTGCGTAATCCATTAATAGCTCGTGCCATTCTTCAAATGTCACTGTTTTTTTAGTGGAACCAAAATCAGCCATATGTATTTCCTGCATTTTGTGGAGGTATCTCTTTGCAAATGAAATCTGAAATATGAACATCAACAGAAAAACACTTTCCTGCTTCTTTTTCTTTTGAGAAGTTTTTTCTGCATTCTTCAGCGGTCATAGGGAAGTCGCTGTAATGAGTCCATTTCACGGGGCCACCACCTGGTTTTTTGATGGTTGCGTTTATTTGGTACTTAGGCATTCAAAAATACCTCCACTCGATCACCTGTCTTTCTTGAACACATCCAGCGGCGCAGTTCTGTAAAACTATCCTGGATCTCACAACGGGTACTTTTCATTGTGACAGTTGGTATATTGTCGTAGCTGGCATATGCAATCTCTTTTTTTGGGGTGCGCAATATGCCTTTGTCAATATCAAGAATCCTGAACGCACTAATTCTTCTTTTGTGCTCGTTAAATAGTGTTCGTTTAGTTGCTGTTATCTGCTTCTGTGCTTCCTGGTAAATGTAGGTAAGCACATCATAAGATTGTTGCTGTGTAATAAGGAAATTACGGCTAACATCCGTTGTTGTGAGCGTTTTATTATTTCTTAATCCCCAATAAGCAACGATGATATAGAGTGGATATCCTTTCAAATCTGCCACACATTCAGGAATGACAGGATTAATTTTTAACTTACGCGCCATATTTTCTCCCGGTTGTTTGTCTGAAATAGAAACATCCCCGGAATACAGCGTCATAAGCTAATTATGAAAAGTTGCAGCCACTCACTACAGGCTCTTTATCTGATTGTTCATCATCATGCATAGCTAAACCCCCTTACCTGGAGTGGAGACTGGCAAATCTGGTGTACTTCTGCGTTGCAGCCTGTACGACTTGCTCAGGGAGAGAGTGAGTGAGATTAACTCAACGCGAGTTCACCAAATAAGGGGGTTTAAGTATGCAGAATGATATTTGCGGATAAAGAGTCGTTACCTGTGTAGATGGCGCGGCACGTCACTCTGTTACGTAGCCCGTTGCTGACAGATGCCAGCGGTGCTTGAAATGCACGAATAATAACCTCCTTATGCCAGGGCTTTAGGCAATGACCGCTGTCCGGGGTTGTTAAAGAGCATGAGATGACAAGCGTTCTAACGGGCGTCAAAGAGTGCTCATTTTCCAGGCAATGAGCACAAAAAACGCGATGAACGCGCTTTTTACAATCATCTGGATAGTATTGTGTGTGCGACATTTAAAACCTGTCAATGCCAGAAGAGCGATCTAATGATCTGTATAATATCTAAAAAACAGAATAATATTCTGTTTGTTAAATGGTGTACGATCTAAAAATCTAGATAATACTGTTTGCGTGGTTTATTCGATCGTGTGAGTGGGTGTATGTAGTCAAAAAAACTTTTTTGAATATACAAAATTCTGAATTTCTGATTTTGTAAGGTTTTGTATGGGGAACGTCAGTAAAGAAGGGGCTTCACTAGCTGTTCTGAAAACGATTTTGAAGCTGTCAGAGATGTGTGTTGAATCAAAAAGGAAACGAGC
